# Prominent Striatum Amyloid Retention in Early-Onset Familial Alzheimer's Disease With PSEN1 Mutations: A Pilot PET/MR Study

**DOI:** 10.3389/fnagi.2021.732159

**Published:** 2021-09-15

**Authors:** Qi Qin, Liping Fu, Ruimin Wang, Jihui Lyu, Huixuan Ma, Minmin Zhan, Aihong Zhou, Fen Wang, Xiumei Zuo, Cuibai Wei

**Affiliations:** ^1^Innovation Center for Neurological Disorders and Department of Neurology, Xuanwu Hospital, National Clinical Research Center for Geriatric Diseases, Capital Medical University, Beijing, China; ^2^Center of Alzheimer's Disease, Beijing Institute for Brain Disorders, Beijing, China; ^3^Beijing Key Laboratory of Geriatric Cognitive Disorders, Beijing, China; ^4^Neurodegenerative Laboratory of Ministry of Education of the People's Republic of China, Beijing, China; ^5^Department of Nuclear Medicine, China-Japan Friendship Hospital, Beijing, China; ^6^Department of Nuclear Medicine, The First Medical Center, Chinese People's Liberation Army General Hospital, Beijing, China; ^7^Center for Cognitive Disorders, Beijing Geriatric Hospital, Beijing, China

**Keywords:** early-onset familial Alzheimer's disease, PET/MR hybrid neuroimaging, amyloid deposition, striatum, Pittsburgh compound-B PET, cognitive performance

## Abstract

**Background:** With the advancements of amyloid imaging in recent years, this new imaging diagnostic method has aroused great interest from researchers. Till now, little is known regarding amyloid deposition specialty in patients with early-onset familial Alzheimer's disease (EOFAD), and even less is known about its role in cognitive impairments.

**Objectives:** Our study aimed to evaluate the amyloid deposition in five patients with EOFAD, 15 patients with late-onset sporadic AD, and 12 healthy subjects utilizing ^11^C-labeled Pittsburgh compound-B (^11^C-PiB) amyloid PET imaging. Moreover, we figured out the correlation between striatal and cortical standardized uptake value ratios (SUVRs). We also investigated the correlation between ^11^C-PiB retention and cognitive presentation.

**Results:** All patients with EOFAD showed high amyloid deposition in the striatum, a pattern that is not usually seen in patients with late-onset sporadic AD. The SUVR in the striatum, especially in the amygdala, showed significant correlations with cortex SUVR in EOFAD. However, neither striatal nor cortical ^11^C-PiB retention was related to cognitive decline.

**Conclusions:** The amyloid distribution in patients with EOFAD differs from late-onset sporadic AD, with higher amyloid deposits in the striatum. Our study also demonstrated positive correlations in ^11^C-PiB retention between the striatum and other cortical areas. We revealed that the distribution of amyloid in the brain is not random but diffuses following the functional and anatomical connections. However, the degree and pattern of amyloid deposition were not correlated with cognitive deficits.

## Introduction

Alzheimer's disease (AD) is the leading cause of dementia and a severe public health problem worldwide (Jia et al., 2019). AD clinical manifestations begin with memory loss and then progress to cognitive dysfunction (Barnett, 2019). Several genetic mutations contribute to AD (Lane et al., 2018). AD is divided into early-onset AD (EOAD) and late-onset AD (LOAD) according to the age of onset (Bird, 2008). LOAD, also known as sporadic AD, is the most common AD with onset age over 65 years (Bateman et al., 2012). The EOAD onset age is earlier than 65 years. Approximately 10% of patients with EOAD are autosomal-dominant inheritance. PSEN1 (HGNC: 9508, OMIM: 104311), PSEN2 (HGNC: 9509, OMIM: 600759), and APP (HGNC: 620, OMIM: 104760) are three primary genes involved in familial EOAD (EOFAD). These genes encode amyloid precursor protein, presenilin-1, and presenilin-2, respectively (Bateman et al., 2011). The PSEN1 mutations are the most prevalent mutations, accounting for 75% of all EOFAD (Qin et al., 2020). The presenilin-1 protein is an essential component of the “y-secretase” enzyme complex, which is responsible for the cleavage of amyloid-β (Aβ) from its precursor APP (Brunkan and Goate, 2005). Therefore, mutations in PSEN1 could result in enhanced amyloid deposition.

The amyloid deposition has been considered as a pathognomonic marker of AD and regarded as an important target of intervention (Hanseeuw et al., 2018). In addition, the National Institute on Aging Alzheimer's Association (NIA-AA) workgroup proposed biomarkers of amyloid levels detected by cerebrospinal fluid assays and PET, which provide feasible tools to diagnose AD (McKhann et al., 2011; Louie, 2019). Moreover, plenty of research studies have indicated that amyloid deposition precedes clinical symptoms. In this regard, early detection of amyloid deposition has emerged as a goal of AD diagnosis and intervention. Thus, the clinical and research utility of amyloid PET imaging has become an effective diagnostic tool for patients with AD and an interesting topic among clinicians and researchers over the years.

The ^11^C-labeled Pittsburgh compound-B (^11^C-PiB) has a high affinity for fibrillar Aβ. This compound was the first ligand used to detect amyloid distribution in AD (Ikonomovic et al., 2008). Typically, the Aβ deposition initiates from the temporal lobe and orbitofrontal cortex and then spreads to the frontal lobe, parietal lobe, precuneus lobe, anterior cortex, and posterior cingulate cortex (Gordon et al., 2018). Over time, not only cortical structures but also subcortical structures can be strongly affected. However, different uptake patterns of early-onset familial carriers deserve special clinical attention. These autosomal-dominant EOFAD gene carriers initiated amyloid deposition in the striatum (Klunk et al., 2007).

In the past few years, hybrid imaging models have been widely accepted in clinical practice. The PET/MRI, a new hybrid model performed better in AD diagnosis. PET imaging can provide metabolic information of the brain, and MRI can provide structural information of the brain. PET/MR can also make up for the deficiency of PET/CT with no ionizing radiation (Arabi and Zaidi, 2016). Thus, the combination of functional imaging (PET) and structural imaging (MRI) has emerged as an accurate technique for AD diagnosis.

Till now, little is known regarding amyloid deposition specialty in patients with EOFAD and even less is known about its role in cognitive dysfunction (Klunk et al., 2007; Villemagne et al., 2009; Cohen et al., 2018). Therefore, our study aimed to use ^11^C-PiB PET/MRI to compare amyloid burden in 5 patients with EOFAD, 15 patients with late-onset sporadic AD, and 12 healthy subjects. Moreover, we investigated the correlation between striatal and cortical standardized uptake value ratios (SUVRs). We also investigated the correlation between ^11^C-PiB retention and clinical and cognitive presentation.

Utilizing amyloid imaging, we aimed to find the difference in amyloid deposition between patients with EOFAD and late-onset sporadic AD. In addition, we intended to reveal the correlation between striatum amyloid and cortex accumulation and the association between amyloid deposition and cognitive presentation.

## Methods

### Subjects

The Institutional Review Board of Xuanwu Hospital approved the study. The methods were in accordance with the Declaration of Helsinki, and each participant signed an informed written consent form.

Five patients with EOFAD and 15 patients with LOAD were recruited from the memory clinic of Xuanwu Hospital, and three senior neurologists diagnosed all patients. The diagnosis met the criteria of the 2018 NIA-AA research framework (Louie, 2019). All participants underwent clinical evaluation, neuropsychological testing, genetic testing, and ^11^C-PiB PET/MRI.

Twelve healthy participants were recruited from the community. They are free from a history of any neurological or psychiatric illness history and served as a normal control (NC) group. Clinical evaluation, neuropsychological testing, genetic testing, and ^11^C-PiB PET/MRI revealed no apparent abnormal findings.

### Neuropsychological Assessment

Neuropsychological evaluations included the Mini-Mental State Examination (MMSE), the Montreal Cognitive Assessment (MoCA), Clock Drawing Test (CDT), the Boston Naming Test (BNT), the Trail Making Test (TMT) A and B, and the Clinical Dementia Rating (CDR) scale assessment.

### Genetic Testing

Genetic testing was performed on DNA obtained from a peripheral blood sample. DNA isolation was extracted from peripheral blood. Exonic regions of early-onset AD genes were captured (MyGenostics GenCap Enrichment Technologies, MyGenostics, Baltimore, MD, USA). The capture experiment was conducted according to the protocol of the manufacturer.

### PET/MRI Procedure

All participants received an ^11^C-PiB PET/MR scan. ^11^C-PiB was synthesized with a radiochemical purity of over 95% and specific activity over 50 GBq/μmol (1.48 Ci/μmol). An initial 40-min intravenous tracer injection (range 333–518 MBq, 0.13–0.15 mCi/kg) was carried out prior to the ^11^C-PiB data acquisition using the Siemens PET/MR systems (Biograph mMR, Siemens Medical Solutions, Grünwald, Germany). The built-in ultrashort echo-time sequence was used for the PET attenuation correction. The PET data were acquired within 20 min. The MRI scanning was performed with the following sequence protocol: sagittal 3D T1WI magnetization-prepared rapid gradient echo (T1WI 3D-MPRAGE): TR = 1,600 ms, TE = 2.15 ms, THK = 1.0 mm, FOV = 256 × 256 mm, matrix = 256 × 256; transverse T2WI fluid-attenuated inversion recovery (T2WI-FLAIR): TR = 8,000 ms, TE = 94 ms, THK = 5 mm, FOV = 192 × 220 mm, flip angle:150°; transverse diffusion-weighted images: with diffusion gradient encoding of *b* = 0, 1,000 s/mm^2^. PET imaging and MRI were performed simultaneously.

### Post-processing

All T1 scans were segmented into the gray matter (GM), white matter (WM), and cerebrospinal fluid (CSF) tissue classes and used the DARTEL group image registration algorithm to build a custom template. Statistical parametric mapping (SPM8) was used to co-register T1 scans with ^11^C-PiB PET scans (http://www.fil.ion.ucl.ac.uk/spm). Co-registered T1 scans were spatially standardized to the custom template and generated the deformation fields for the ^11^C-PiB PET scans, respectively. The cerebellar gray matter (CGM) region mask was created from the automated anatomic labeling (AAL) atlas and the GM mask. In order to obtain the individual CGM region, the AAL atlas was first transformed to the custom template space and then inverse transformed to the individual ^11^C-PiB space using the inverse transformation of the deformation field obtained. The same normalization procedure was applied to the other 90 region of interests (ROIs) derived from the AAL atlas to obtain the individual ROIs. The CGM was selected as the reference region for SUVR measurement.

### Statistical Analysis

Statistical data analysis was performed using the SPSS software (Version 20, SPSS Inc., Chicago, IL, USA). The assessment criterion of demographic information and neuropsychological tests between the EOFAD, LOAD, and NC were performed with one-way ANOVA. The SUVR of ROIs between groups was compared with one-way ANOVA at *p* < 0.05. Partial correlation analyses controlling for age and sex were used to study the relationship between SUVR value and neuropsychological test scores (*p* < 0.05, Bonferroni corrected, *N* = the statistical subject numbers).

## Results

### Demographics and Clinical Characterization for EOFAD

Table 1 displays demographic, neuropsychological, genetic, and leading clinical symptoms for EOFAD. Among all these five patients, four of them are female. The average age of onset is 38.6 years old. All patients display progressive memory decline as the first symptom, especially working memory impairment. Besides memory loss, four patients exhibit decreased executive function. In addition, all patients with EOFAD showed significantly decreased cognitive scores on calculation and visuospatial function. None of the patients showed aphasia. Except for cognitive decline, the neurological examination reveals a positive Babinski sign and slightly increased muscular tension in Case 3. Case 4 has a positive family history. Notably, four patients suffered behavioral and psychological symptoms, such as depression and anxiety. The results of MMSE, MoCA, CDT, TMT A and B, BNT CDR, and the neuropsychiatric inventory (NPI) assessment were listed in Table 1. Initial blood chemistry and cerebral spinal fluid (CSF) analyses are negative. Five patients with early-onset AD undertook genetic testing, and all of them carry mutations in PSEN1.

**Table 1:** Demographics and clinical symptoms of five patients with early-onset familial Alzheimer's disease (EOFAD).

**Case**		**1**	**2**	**3**	**4**	**5**
Sex		Female	Female	Male	Female	Female
Onset age (years)		36	39	40	43	35
Education (years)		9	9	16	15	12
Cognitive impartment	Memory impairment	+	+	+	+	+
	Executive impairment	+	+	—	+	+
	Calculation impairment	+	+	+	+	+
	Visuospatial impairment	+	+	+	+	+
	Aphasia	—	—	—	—	—
Other neurological presentation		—	—	Positive Babinski sign Increased muscular tension	—	—
Depression or anxiety		+	—	+	+	+
Family history		—	+	—	+	—
Neuro-psychological test	MMSE	13	19	10	21	15
	MoCA	8	13	6	18	13
	CDT	1	2	0	2	1
	BNT	13	18	8	22	17
	TMT A	135	73.8	97.6	63.2	150
	TMT B	300	106	175	94	204
	CDR	2	1	2	1	2
	NPI	46	17	36	22	35
Genetic mutation		PSEN1	PSEN1	PSEN1	PSEN1	PSEN1
		C410Y	L173F	G206S	L219P	F177L

*BHT, Boston Naming Test; CDR, Clinical Dementia Rating; CDT, Clock Drawing Test; MMSE, Mini-Mental State Examination; MoCA, Montreal Cognitive Assessment; NPI, neuropsychiatric inventory; TMT, Trail Making Test*.

### Pattern of ^11^C-PiB PET/MR Distribution in EOFAD, LOAD, and NC Groups

**Figure 1:**
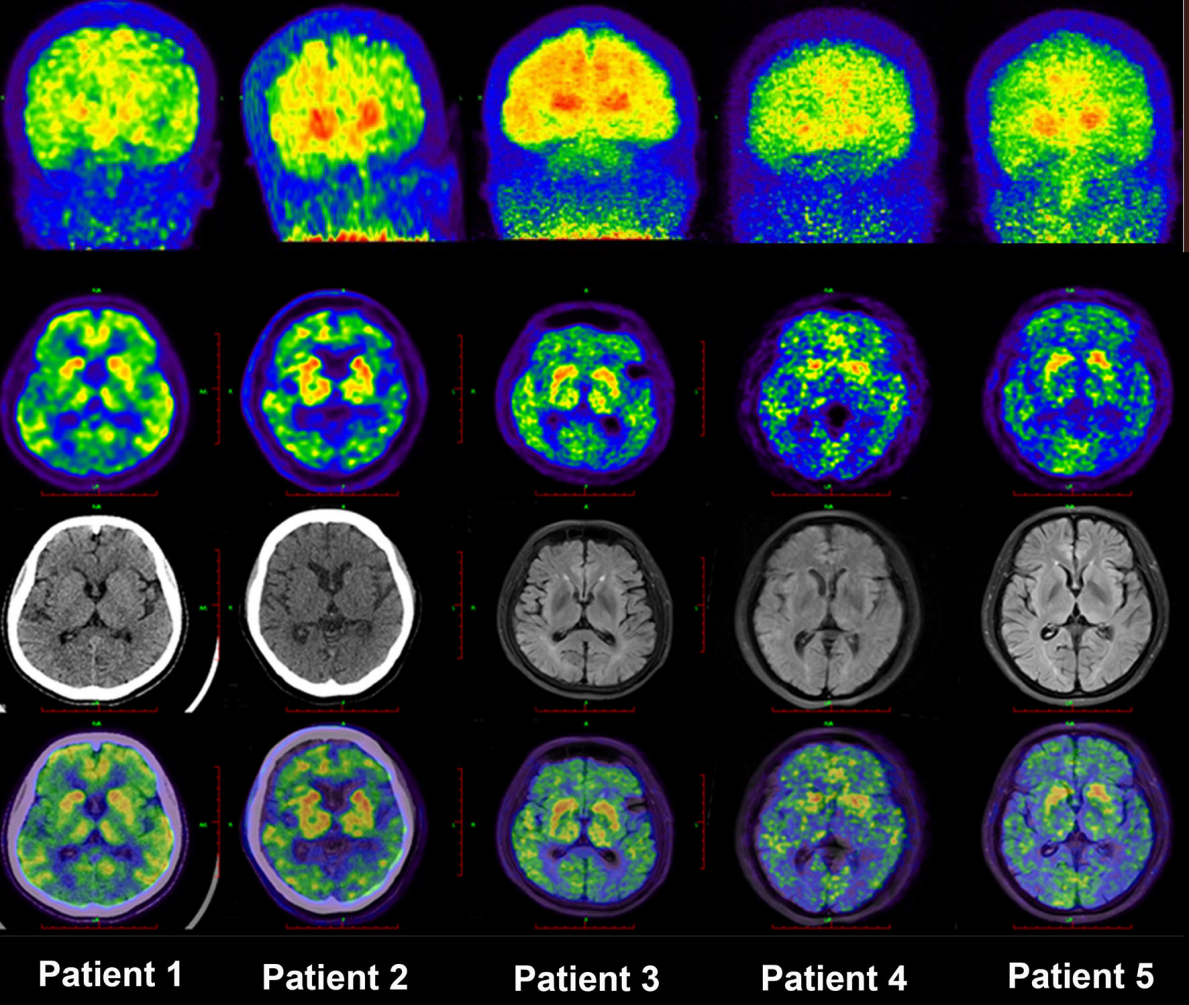
MIP images of PIB-PET in five patients with EOFAD. The maximum intensity projection images of ^11^C-PIB PET in five patients with EOFAD were shown, and increased PIB retention was detected in both the neocortex and striatum. ^11^C-PiB, ^11^C-labeled Pittsburgh compound-B; EOFAD, early-onset familial Alzheimer's disease.

**Figure 2:**
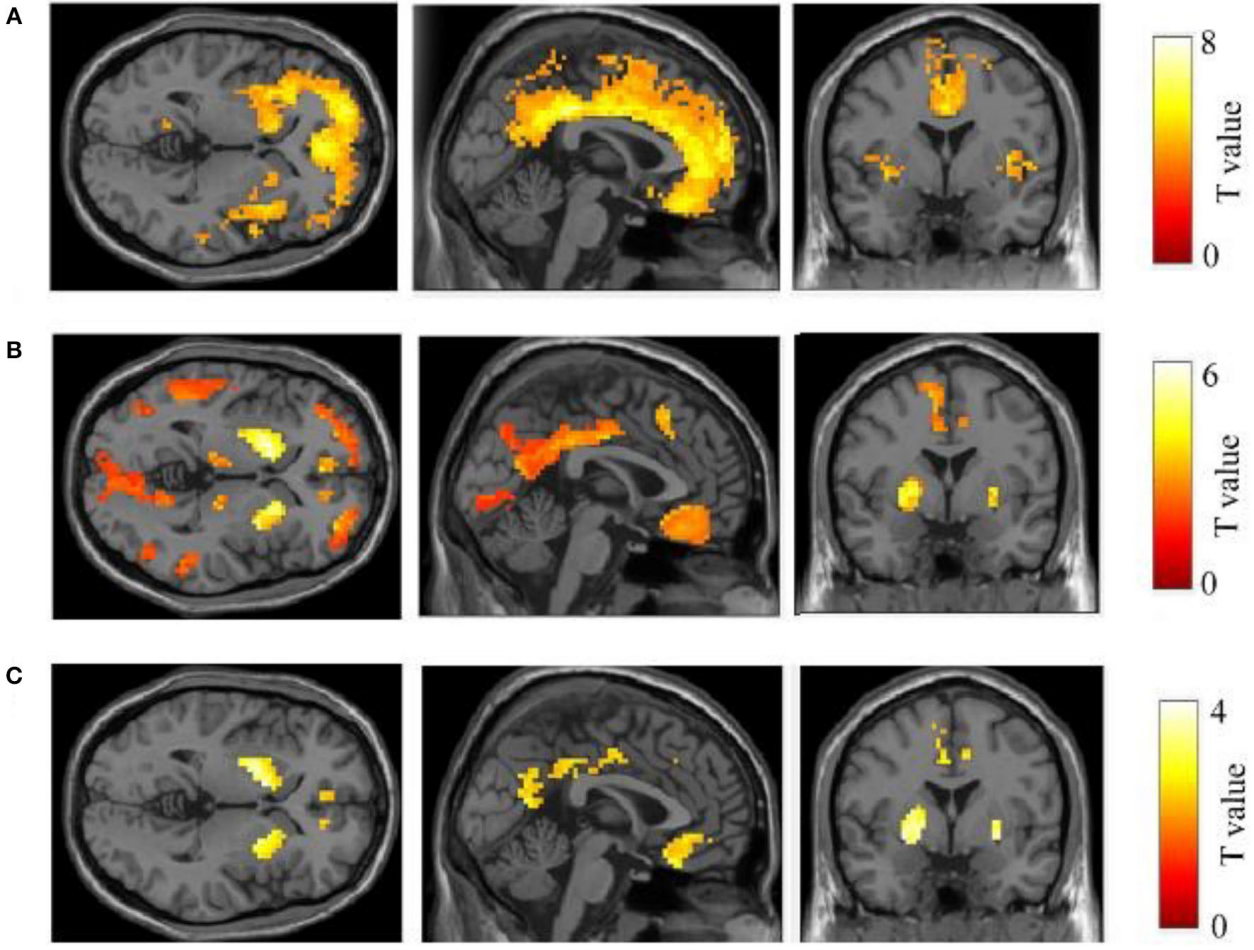
Comparison of ^11^C-PIB retention between three groups. In the comparison between LOAD–NC **(A)** and EOFAD–NC **(B)**, both AD groups were observed greater ^11^C-PIB retention in the cortical and striatal regions. But the caudate, putamen, and amygdala were shown greater ^11^C-PIB accumulation in patients with EOFAD than that in patients with LOAD **(C)**. ^11^C-PiB, ^11^C-labeled Pittsburgh compound-B; EOFAD, early-onset familial Alzheimer's disease; LOAD, late-onset Alzheimer's disease.

**Figure 3:**
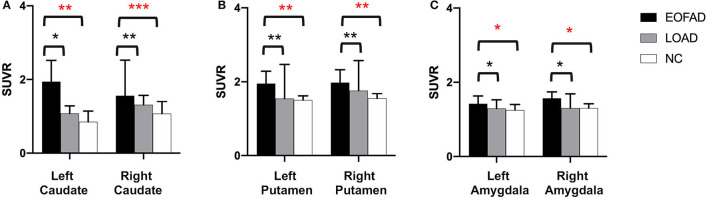
^11^C-PIB retention among patients with EOFAD, patients with LOAD, and NC group in the striatum. There were significant SUVR differences among the three groups in caudate **(A)**, putamen **(B)**, and amygdala **(C)**. ^11^C-PiB, ^11^C-labeled Pittsburgh compound-B; EOFAD, early-onset familial Alzheimer's disease; LOAD, late-onset Alzheimer's disease; NC, normal control; SUVR, standardized uptake value ratio. *means *P* < 0.05, **means *P* < 0.01, and ***p < 0.001.

The ^**11**^**C**-PiB PET/MR images of five patients with FAD, 15 patients with late-onset sporadic AD, and 12 NC subjects with the definite clinical diagnoses were analyzed. As expected, all the patients with EOFAD and late-onset sporadic AD performed significantly worse on MMSE compared with NC subjects (Supplementary Table 1). The maximum intensity projection images of ^11^C-PiB PET in five patients with EOFAD were shown in Figure 1, in which increased ^11^C-PiB retention was detected in both neocortex and striatum. From the ROI analysis, there were significant SUVR differences among the three groups in the frontal cortex, precuneus, anterior cingulated cortex, and parietal lobe (Figures 2, 3 and Supplementary Table 2). When compared to patients with EOFAD than that of patients with LOAD, the ^11^C-PiB retention rate was found with no significant increase in 29 ROIs, but both two AD groups showed higher SUVR retention than the NC group. Regarding the three ROIs of the striatum, including caudate (*p* = 0.014, *p* = 0.015), putamen (*p* = 0.012, *p* = 0.008), and amygdala (*p* = 0.016, *p* = 0.02), the ^11^C-PiB retention of EOFAD was found significantly higher than LOAD and NC (Supplementary Tables 3, 4).

### Correlations of ^11^C-PiB Accumulations Between Striatum and Cortex in EOFAD

Comparisons of ^11^C-PiB distribution revealed differences among three groups, with higher striatal uptake in patients with EOFAD. Then, we detected correlations of ^11^C-PiB accumulations between striatum and cortex in EOFAD. The strongest correlation with striatal uptake was seen in the amygdala. The amygdala ^11^C-PiB accumulations significantly correlated with majority cortex ROIs including precentral area, frontal lobe, rolandic operculum, supplementary motor area, olfactory, rectus, insula, cingulate gyrus, hippocampus, parahippocampal area, calcaneus, cuneus, lingual area, occipital area, fusiform area, postcentral area, parietal area, supralimbic area, angular, precuneus area, paracentral lobule, globus pallidus, Heschl's gyrus, and temporal lobe. The correlation between the amygdala and frontal lobe ^11^C-PiB accumulations is the most significant (*F* = 15.659*, p* = 0.001). The ^11^C-PiB uptake of caudate also correlated significantly with several cortex areas such as rolandic operculum, insula, calcarine, cuneus, lingual, occipital lobe, posterior central lobe, parietal lobe, marginal superior horn lobe, paracentral lobule, and thalamus pallidus (Supplementary Table 5). These results revealed strong correlations of ^11^C-PiB accumulations between striatum and cortex in EOFAD. They indicated that the ^11^C-PIB accumulations in the striatum in patients with EOFAD are associated with amyloid accumulations in the cortex.

### Correlations Between ^11^C-PiB Accumulations and Neuropsychological Test in EOFAD

Besides ^11^C-PiB accumulations of ROIs in EOFAD, we also detected the correlation of ^11^C-PiB accumulations and neuropsychological tests in EOFAD. There was no significant difference between ^11^C-PiB retention and neuropsychological test. Therefore, amyloid distribution in patients with EOFAD was not associated with cognitive impairment (Supplementary Table 6).

## Discussion

The ^11^C-PiB amyloid PET/MRI provides a new perspective on Aβ deposition in the brain, and this auxiliary examination method facilitates research into the etiology, diagnosis, and treatment of AD (Linazasoro, 2008). Our study examined the pattern and degree of ^11^C-PiB retention in five familial AD cases with PSEN1 mutations. All PSEN1 mutation carriers showed increased ^11^C-PiB retention. Although the degree of cortical retention was lower than patients with late-onset sporadic AD, the striatal retention was remarkably higher. Furthermore, the high degree of striatum ^11^C-PiB retention in patients with EOFAD is difficult to coincide than with patients with late-onset sporadic AD with the same clinical symptoms, and this pattern is coincident in previously reported patients with EOFAD (Klunk et al., 2007; Villemagne et al., 2009; Blautzik et al., 2017). Postmortem studies of patients with PSEN1 mutation also showed Aβ deposition in the striatum (Villemagne et al., 2009). These EOFAD studies suggested that amyloid deposition may follow a specific order, beginning in the striatum and then spreading diffusely throughout the neocortex (Klunk et al., 2007; Villemagne et al., 2009). There are four possible underlying mechanisms on the relatively early involvement of the striatum. First, the cortical predominantly amyloid deposition in patients with late-onset sporadic AD may be influenced by synaptic processes, whereas in EOFAD, amyloid deposition in subcortical areas, such as the striatum, may be mediated by amyloid precursor protein and its processing (Koivunen et al., 2008; Ishibashi et al., 2014). Second, the APP processing patterns differed between patients with EOFAD and sporadic AD. The PSEN1 gene mutation could induce axonal mis-trafficking, which was suggested as a potential culprit for striatal amyloid deposition. Such axonal mis-trafficking is considered to stem from a disruption in the APP processing (Maeda et al., 2007). Moreover, the striatum is vulnerable to tau protein accumulation in familial AD in the early stage (Jack et al., 2013a). Tau accumulation is considered more toxic to induce significant striatal neuronal injury. In addition, other studies concluded that different amyloid deposition could be due to different susceptibility to amyloidosis (Blautzik et al., 2017). The striatum exhibits amyloid deposition only in a more advanced phase of amyloidosis (Teipel et al., 2020). Patients with EOFAD showed more advanced amyloidosis in the striatum.

Given this background, our study also set out to investigate the connections between striatal and cortical regions. We detected that the ^11^C-PiB accumulation in the striatum is correlated with cortex ^11^C-PiB accumulation, especially in the amygdala. A similar study by Ishibashi et al. (2014) also found the highest ^11^C-PiB distribution in the ventral striatum, and the SUVR value strongly correlated with ^11^C-PiB retention in the frontal area (Koivunen et al., 2008). Our study also found that the correlations occurred between the amygdala and frontal lobe accumulation are the most significant. However, the connections between the frontal cortex and striatum are still not fully understood. Several studies have shown an anterograde distribution of input neurons in areas affected by amyloid deposition (Ikonomovic et al., 2008; Jung et al., 2010). In contrast to this finding, the striatum receives inputs from the frontal cortex, as revealed by a previous rat study (Mehlman et al., 2019). According to this study, the frontal cortex plays a vital role in determining the biological significance of associative information, and the input of the hippocampus may not be filtered. Moreover, several studies pointed out that the frontal lobe and the striatum are essential for executive function and decision-making (Seok et al., 2015; Orr et al., 2019). Therefore, the significant correlation between the striatum and cortical areas may explain the decision-making dysfunction in patients with EOFAD. There was also a high correlation between the cingulate cortex and striatum in our study. A previous study found significant functional connections between the cingulate cortex and striatum in patients with depression. The cingulate cortex showed reduced glucose metabolism in patients with depression, so it is assumed to be susceptible to depression (Clery-Melin et al., 2019). In our study, patients with EOFAD always presented with depression (four of five patients had depression or anxiety as shown in Figure 1). This further reinforces our reasoning that the strong correlation between cortical amyloid deposition and striatal amyloid accumulation is not random but reflects the functional connections.

Moreover, we found a significant negative correlation between cognitive performance and amyloid accumulation. This may be due to the progress of a biomarker, such as ^11^C-PiB PET showed no linear increase in amyloid protein and cognitive decline (Tentolouris-Piperas et al., 2017). The ^11^C-PiB positive cases in our study may have already presented amyloid accumulation, so the neuropsychological test showed no further correlation between amyloid disposition and cognitive decline. Moreover, epidemiologic evidence suggests that cognitive decline in AD is also affected by other protective factors, such as cognitive training, high IQ, and high levels of education (Vemuri et al., 2011). Furthermore, we detect that the intense and focal striatal amyloid deposition in EOFAD did not lead to any movement disorder. This phenomenon indicated that striatum is not essential for the movement but is involved in decision-making in EOFAD.

Our study detected higher striatal ^11^C-PiB retention in patients with EOFAD with PSEN1 mutations, unlike in patients with late-onset sporadic AD. The pattern and extent of Aβ accumulation were not associated with cognitive decline. Nevertheless, the distribution of amyloid deposits in the striatum correlated with the accumulation of cortical ^11^C-PiB, particularly in the amygdala.

There are also some limitations of our study. First of all, our study is a cross-sectional study. Longitudinal and therapeutic studies are needed to compare striatal and neocortical ^11^C-PiB measurements to track Aβ plaque deposition, evaluate AD treatments, and prognoses in patients with EOFAD. Then, there are only five cases included in our study; additional cases are needed to draw the conclusion. In addition, studies are needed to clarify the species and range of Aβ species merge to Aβ-tracer. ^11^C-PiB shows higher affinity to N-terminal-truncated Aβ42 species in senile plaques and is less sensitive to diffuse Aβ plaques (Jack et al., 2013b). Cotton wool plaques are composed mainly of Aβ42 species and can be observed in the striatum of PSEN1 mutation carriers (Miki et al., 2019). More extensive studies should be conducted on cotton wool plaques to explain their etiological mechanisms and how they lead to different patterns of ^11^C-PiB retention among patients with EOFAD and late-onset sporadic AD, and normal controls.

## Conclusions

The amyloid deposition in EOFAD differs from that in late-onset sporadic AD; in that, the striatal ^11^C-PiB retention is higher in EOFAD. Our study also found that amyloid deposition in the striatum correlated with the accumulation of cortical ^11^C-PiB, particularly in the amygdala. The significant correlation between striatal and cortical areas is not random but reflects a functional link. Furthermore, the pattern and extent of amyloid distribution did not correlate with cognitive status.

## Data Availability Statement

The raw data supporting the conclusions of this article will be made available by the authors, without undue reservation.

## Ethics Statement

The studies involving human participants were reviewed and approved by Xuanwu Hospital affiliated to Capital Medical University. The patients/participants provided their written informed consent to participate in this study. Written informed consent was obtained from the individual(s) for the publication of any potentially identifiable images or data included in this article.

## Author Contributions

CW, HM, and MZ collected the clinical data of patients and conducted neuropsychological tests. LF, RW, and QQ acquired the PET/MRI and executed post-processing. JL analyzed genetic reports. QQ performed the statics analysis. AZ, FW, and XZ participated in the literature review and helped to draft the manuscript. QQ drafted the manuscript. LF and CW revised the article. All authors read and approved the final manuscript.

## Funding

This study was supported by the National Key R&D Program of China (No. 2017YFC1310103), Capital's Funds for Health Improvement and Research (CFH2020-4-1033), Beijing Municipal Administration of Hospitals Clinical Medicine Development of Special Funding Support (No. ZYLX201837), National Key R&D Program (2016YFC1306305), and Beijing Municipal Natural Science Foundation (No. 7192192).

## Conflict of Interest

The authors declare that the research was conducted in the absence of any commercial or financial relationships that could be construed as a potential conflict of interest. The reviewer JY declared a shared affiliation, though no other collaboration, with several of the authors QQ, HM, MZ, AZ, FW, XZ, and CW to the handling editor.

## Publisher's Note

All claims expressed in this article are solely those of the authors and do not necessarily represent those of their affiliated organizations, or those of the publisher, the editors and the reviewers. Any product that may be evaluated in this article, or claim that may be made by its manufacturer, is not guaranteed or endorsed by the publisher.
