# Supplementary material for: Prominent Striatum Amyloid Retention in Early-Onset Familial Alzheimer's Disease With PSEN1 Mutations: A Pilot PET/MR Study
Source: Front Aging Neurosci. 2021 Sep 15;13:732159. doi: 10.3389/fnagi.2021.732159 (PMC8480470; doi:10.3389/fnagi.2021.732159)
Supplement: Supplementary file 1 [file Table_1.DOCX]

**Supplementary Table 1. Demographics and neuropsychological tests between EOFAD, LOAD and NC.**

|  | **EOFAD (N=5)** | **LOAD (N=15)** | **NC (N=12)** | **P value** |
| --- | --- | --- | --- | --- |
| **Sex (male/female)** | 1/4 | 7/8 | 6/6 | 0.734 |
| **Age** | 38.8± 3.3 | 62.7± 2.8 | 66.4± 5.9 | 2.667E-9*** |
| **MMSE** | 14.7± 1.5 | 22.3± 3.1 | 29.4± 0.9 | 9.192E-5*** |

**Supplementary Table 2. SUVR of EOFAD, LOAD and NC groups on entire 90 ROIs.**

|  | **EOFAD** | | **LOAD** | | **NC** | | **T** | **P** |
| --- | --- | --- | --- | --- | --- | --- | --- | --- |
|  | **Mean** | **SD** | **Mean** | **SD** | **Mean** | **SD** |  |  |
| **Precentral_L2001** | 1.303 | 0.769 | 1.562 | 0.257 | 1.309 | 0.139 | 2.027 | 0.151 |
| **Precentral_R2002** | 1.336 | 0.773 | 1.491 | 0.250 | 1.261 | 0.100 | 1.451 | 0.251 |
| **Frontal_Sup_L2101** | 1.699 | 0.597 | 1.806 | 0.252 | 1.298 | 0.193 | 8.822 | 0.001*** |
| **Frontal_Sup_R2102** | 1.683 | 0.569 | 1.686 | 0.268 | 1.231 | 0.203 | 7.545 | 0.002** |
| **Frontal_Sup_Orb_L2111** | 1.967 | 0.753 | 2.093 | 0.282 | 1.484 | 0.207 | 8.936 | 0.001*** |
| **Frontal_Sup_Orb_R2112** | 1.968 | 0.726 | 1.972 | 0.381 | 1.418 | 0.232 | 6.508 | 0.005** |
| **Frontal_Mid_L2201** | 1.855 | 0.474 | 1.834 | 0.261 | 1.317 | 0.215 | 11.727 | 2.112E-5*** |
| **Frontal_Mid_R2202** | 1.540 | 0.663 | 1.600 | 0.282 | 1.142 | 0.200 | 6.001 | 0.007** |
| **Frontal_Mid_Orb_L2211** | 1.903 | 0.792 | 2.107 | 0.242 | 1.462 | 0.216 | 9.806 | 0.001*** |
| **Frontal_Mid_Orb_R2212** | 1.942 | 0.578 | 1.915 | 0.363 | 1.318 | 0.244 | 9.588 | 0.001*** |
| **Frontal_Inf_Oper_L2301** | 1.563 | 0.965 | 1.710 | 0.252 | 1.337 | 0.176 | 2.509 | 0.099 |
| **Frontal_Inf_Oper_R2302** | 1.541 | 0.927 | 1.642 | 0.223 | 1.324 | 0.161 | 2.062 | 0.146 |
| **Frontal_Inf_Tri_L2311** | 1.761 | 0.811 | 1.844 | 0.214 | 1.405 | 0.176 | 4.949 | 0.014* |
| **Frontal_Inf_Tri_R2312** | 1.552 | 0.948 | 1.676 | 0.275 | 1.268 | 0.174 | 3.013 | 0.065 |
| **Frontal_Inf_Orb_L2321** | 1.617 | 0.953 | 1.745 | 0.185 | 1.281 | 0.160 | 4.451 | 0.021* |
| **Frontal_Inf_Orb_R2322** | 1.564 | 0.921 | 1.616 | 0.263 | 1.212 | 0.197 | 3.229 | 0.055 |
| **Rolandic_Oper_L2331** | 1.595 | 1.045 | 1.621 | 0.312 | 1.244 | 0.191 | 2.239 | 0.125 |
| **Rolandic_Oper_R2332** | 1.604 | 0.997 | 1.659 | 0.259 | 1.319 | 0.112 | 2.130 | 0.138 |
| **Supp_Motor_Area_L2401** | 1.721 | 0.372 | 1.618 | 0.282 | 1.124 | 0.163 | 14.183 | 5.572E-5*** |
| **Supp_Motor_Area_R2402** | 1.712 | 0.665 | 1.713 | 0.285 | 1.292 | 0.166 | 5.537 | 0.009** |
| **OIfactory_L2501** | 1.569 | 1.027 | 1.453 | 0.249 | 1.123 | 0.149 | 2.539 | 0.097 |
| **OIfactory_R2502** | 1.531 | 1.034 | 1.435 | 0.202 | 1.139 | 0.150 | 2.094 | 0.142 |
| **Frontal_Sup_Medial_L2601** | 1.878 | 0.488 | 1.753 | 0.258 | 1.155 | 0.218 | 17.004 | 1.466E-5*** |
| **Frontal_Sup_Medial_R2602** | 1.757 | 0.629 | 1.717 | 0.273 | 1.177 | 0.218 | 9.723 | 0.001*** |
| **Frontal_Mid_Orb_L2611** | 2.297 | 0.546 | 1.996 | 0.356 | 1.285 | 0.276 | 17.740 | 1.055E-5*** |
| **Frontal_Mid_Orb_R2612** | 2.209 | 0.619 | 2.003 | 0.356 | 1.401 | 0.268 | 11.072 | 2.866E-5*** |
| **Rectus_L2701** | 2.341 | 0.558 | 1.918 | 0.355 | 1.258 | 0.269 | 18.131 | 8.887E-6*** |
| **Rectus_R2702** | 2.143 | 0.691 | 1.945 | 0.362 | 1.349 | 0.275 | 9.651 | 0.001*** |
| **Insula_L3001** | 1.585 | 1.027 | 1.675 | 0.227 | 1.292 | 0.116 | 2.637 | 0.089 |
| **Insula_R3002** | 1.613 | 1.017 | 1.665 | 0.233 | 1.319 | 0.132 | 2.196 | 0.130 |
| **Cingulum_Ant_L4001** | 2.070 | 0.655 | 1.887 | 0.283 | 1.366 | 0.091 | 11.554 | 2.191E-5*** |
| **Cingulum_Ant_R4002** | 2.095 | 0.643 | 1.941 | 0.304 | 1.470 | 0.113 | 8.772 | 0.001*** |
| **Cingulum_Mid_L4011** | 2.350 | 0.539 | 2.080 | 0.277 | 1.430 | 0.158 | 22.059 | 1.768E-6*** |
| **Cingulum_Mid_R4012** | 2.211 | 0.518 | 2.041 | 0.260 | 1.541 | 0.151 | 13.700 | 7.096E-5*** |
| **Cingulum_Post_L4021** | 1.899 | 0.465 | 1.908 | 0.204 | 1.503 | 0.167 | 9.335 | 0.001*** |
| **Cingulum_Post_R4022** | 1.615 | 0.541 | 1.829 | 0.233 | 1.574 | 0.160 | 2.948 | 0.069 |
| **Hippocampus_L4101** | 1.177 | 0.746 | 1.368 | 0.193 | 1.349 | 0.105 | 0.697 | 0.507 |
| **Hippocampus_R4102** | 1.188 | 0.716 | 1.374 | 0.224 | 1.431 | 0.091 | 1.021 | 0.373 |
| **ParaHippocampal_L4111** | 1.059 | 0.640 | 1.304 | 0.146 | 1.203 | 0.118 | 1.600 | 0.220 |
| **ParaHippocampal_R4112** | 1.160 | 0.683 | 1.341 | 0.175 | 1.279 | 0.113 | 0.726 | 0.493 |
| **Amygdala_L4201** | 1.422 | 0.209 | 1.29 | 0.236 | 1.253 | 0.149 | 0.119 | 0.888 |
| **Amygdala_R4202** | 1.567 | 0.176 | 1.303 | 0.382 | 1.304 | 0.117 | 0.090 | 0.915 |
| **Calcarine_L5001** | 1.537 | 0.577 | 1.507 | 0.295 | 1.290 | 0.203 | 1.713 | 0.199 |
| **Calcarine_R5002** | 1.607 | 0.618 | 1.538 | 0.309 | 1.397 | 0.191 | 0.849 | 0.439 |
| **Cuneus_L5011** | 1.320 | 0.774 | 1.525 | 0.321 | 1.367 | 0.184 | 0.795 | 0.461 |
| **Cuneus_R5012** | 1.297 | 0.765 | 1.467 | 0.310 | 1.350 | 0.192 | 0.514 | 0.604 |
| **Lingual_L5021** | 1.284 | 0.510 | 1.424 | 0.220 | 1.259 | 0.180 | 1.317 | 0.284 |
| **Lingual_R5022** | 1.340 | 0.327 | 1.381 | 0.222 | 1.254 | 0.142 | 1.088 | 0.351 |
| **Occipital_Sup_L5101** | 1.237 | 0.693 | 1.483 | 0.306 | 1.336 | 0.176 | 1.100 | 0.347 |
| **Occipital_Sup_R5102** | 1.179 | 0.703 | 1.412 | 0.294 | 1.292 | 0.193 | 0.904 | 0.416 |
| **Occipital_Mid_L5201** | 1.353 | 0.747 | 1.561 | 0.299 | 1.293 | 0.220 | 1.742 | 0.194 |
| **Occipital_Mid_R5202** | 1.210 | 0.722 | 1.409 | 0.298 | 1.208 | 0.216 | 1.145 | 0.333 |
| **Occipital_Inf_L5301** | 1.341 | 0.644 | 1.565 | 0.315 | 1.290 | 0.255 | 2.004 | 0.154 |
| **Occipital_Inf_R5302** | 1.222 | 0.640 | 1.376 | 0.275 | 1.234 | 0.197 | 0.745 | 0.484 |
| **Fusiform_L5401** | 1.273 | 0.697 | 1.441 | 0.215 | 1.205 | 0.170 | 1.819 | 0.181 |
| **Fusiform_R5402** | 1.373 | 0.750 | 1.489 | 0.220 | 1.313 | 0.170 | 0.891 | 0.421 |
| **Postcentral_L6001** | 1.295 | 0.816 | 1.546 | 0.280 | 1.196 | 0.164 | 2.850 | 0.075 |
| **Postcentral_R6002** | 1.275 | 0.774 | 1.488 | 0.257 | 1.189 | 0.165 | 2.341 | 0.115 |
| **Parietal_Sup_L6101** | 1.362 | 0.874 | 1.606 | 0.321 | 1.195 | 0.246 | 3.006 | 0.066 |
| **Parietal_Sup_R6102** | 1.212 | 0.764 | 1.415 | 0.302 | 1.063 | 0.240 | 2.671 | 0.087 |
| **Parietal_Inf_L6201** | 1.477 | 0.900 | 1.739 | 0.308 | 1.248 | 0.246 | 4.174 | 0.026* |
| **Parietal_Inf_R6202** | 1.303 | 0.769 | 1.630 | 0.313 | 1.168 | 0.247 | 3.900 | 0.032* |
| **SupraMarginal_L6211** | 1.336 | 0.773 | 1.708 | 0.303 | 1.251 | 0.235 | 3.587 | 0.041* |
| **SupraMarginal_R6212** | 1.699 | 0.597 | 1.654 | 0.274 | 1.249 | 0.142 | 3.738 | 0.036* |
| **Angular_L6221** | 1.683 | 0.569 | 1.782 | 0.304 | 1.346 | 0.270 | 3.041 | 0.064 |
| **Angular_R6222** | 1.967 | 0.753 | 1.657 | 0.275 | 1.298 | 0.235 | 2.401 | 0.109 |
| **Precuneus_L6301** | 1.968 | 0.726 | 1.850 | 0.265 | 1.310 | 0.264 | 7.793 | 0.002*** |
| **Precuneus_R6302** | 1.855 | 0.474 | 1.857 | 0.259 | 1.371 | 0.286 | 7.028 | 0.003*** |
| **Paracentral_Lobule_L6401** | 1.540 | 0.663 | 1.578 | 0.264 | 1.229 | 0.173 | 2.929 | 0.070 |
| **Paracentral_Lobule_R6402** | 1.903 | 0.792 | 1.663 | 0.261 | 1.276 | 0.218 | 2.902 | 0.072 |
| **Caudate_L7001** | 1.942 | 0.578 | 1.079 | 0.204 | 0.852 | 0.291 | 2.357 | 0.113 |
| **Caudate_R7002** | 1.563 | 0.965 | 1.311 | 0.261 | 1.073 | 0.328 | 1.831 | 0.179 |
| **Putamen_L7011** | 1.951 | 0.334 | 1.541 | 0.927 | 1.502 | 0.112 | 24.746 | 6.466E-7*** |
| **Putamen_R7012** | 1.978 | 0.346 | 1.761 | 0.811 | 1.547 | 0.133 | 11.788 | 1.932E-5*** |
| **Pallidum_L7021** | 1.552 | 0.948 | 1.954 | 0.267 | 1.750 | 0.138 | 4.414 | 0.022** |
| **Pallidum_R7022** | 1.617 | 0.953 | 1.970 | 0.260 | 1.762 | 0.124 | 1.385 | 0.267 |
| **Thalamus_L7101** | 1.564 | 0.921 | 1.450 | 0.151 | 1.298 | 0.151 | 1.406 | 0.262 |
| **Thalamus_R7102** | 1.595 | 1.045 | 1.481 | 0.181 | 1.376 | 0.148 | 0.438 | 0.650 |
| **Heschl_L8101** | 1.604 | 0.997 | 1.671 | 0.323 | 1.363 | 0.160 | 1.321 | 0.283 |
| **Heschl_R8102** | 1.721 | 0.372 | 1.756 | 0.294 | 1.471 | 0.107 | 1.183 | 0.321 |
| **Temporal_Sup_L8111** | 1.712 | 0.665 | 1.642 | 0.301 | 1.280 | 0.190 | 2.509 | 0.099 |
| **Temporal_Sup_R8112** | 1.569 | 1.027 | 1.699 | 0.286 | 1.347 | 0.168 | 2.128 | 0.138 |
| **Temporal_Pole_Sup_L8121** | 1.531 | 1.034 | 1.283 | 0.220 | 1.080 | 0.133 | 1.411 | 0.261 |
| **Temporal_Pole_Sup_R8122** | 1.878 | 0.488 | 1.327 | 0.227 | 1.132 | 0.157 | 1.051 | 0.363 |
| **Temporal_Mid_L8201** | 1.757 | 0.629 | 1.737 | 0.301 | 1.355 | 0.233 | 4.450 | 0.021** |
| **Temporal_Mid_R8202** | 2.297 | 0.546 | 1.674 | 0.272 | 1.339 | 0.211 | 2.729 | 0.083 |
| **Temporal_Pole_Mid_L8211** | 2.209 | 0.619 | 1.443 | 0.239 | 1.199 | 0.156 | 2.815 | 0.077 |
| **Temporal_Pole_Mid_R8212** | 2.341 | 0.558 | 1.452 | 0.239 | 1.212 | 0.150 | 2.477 | 0.102 |
| **Temporal_Inf_L8301** | 2.143 | 0.691 | 1.447 | 0.320 | 1.117 | 0.218 | 2.717 | 0.083 |
| **Temporal_Inf_R8302** | 1.585 | 1.027 | 1.461 | 0.280 | 1.219 | 0.197 | 1.637 | 0.213 |

**Supplementary Table 3. SUVR between EOFAD and LOAD groups on entire 90 ROIs.**

|  | **EOFAD** | | | **LOAD** | | **T** | **P** |
| --- | --- | --- | --- | --- | --- | --- | --- |
|  | **Mean** | **SD** | **Mean** | | **SD** |  |  |
| **Precentral_L2001** | 1.303 | 0.769 | 1.562 | | 0.257 | 7.096 | 0.016** |
| **Precentral_R2002** | 1.336 | 0.773 | 1.491 | | 0.25 | 6.162 | 0.023** |
| **Frontal_Sup_L2101** | 1.699 | 0.597 | 1.806 | | 0.252 | 7.281 | 0.015** |
| **Frontal_Sup_R2102** | 1.683 | 0.569 | 1.686 | | 0.268 | 2.666 | 0.12 |
| **Frontal_Sup_Orb_L2111** | 1.967 | 0.753 | 2.093 | | 0.282 | 5.701 | 0.028** |
| **Frontal_Sup_Orb_R2112** | 1.968 | 0.726 | 1.972 | | 0.381 | 1.694 | 0.21 |
| **Frontal_Mid_L2201** | 1.855 | 0.474 | 1.834 | | 0.261 | 3.983 | 0.061 |
| **Frontal_Mid_R2202** | 1.54 | 0.663 | 1.6 | | 0.282 | 4.319 | 0.052 |
| **Frontal_Mid_Orb_L2211** | 1.903 | 0.792 | 2.107 | | 0.242 | 15.659 | 0.001** |
| **Frontal_Mid_Orb_R2212** | 1.942 | 0.578 | 1.915 | | 0.363 | 0.569 | 0.46 |
| **Frontal_Inf_Oper_L2301** | 1.563 | 0.965 | 1.71 | | 0.252 | 7.144 | 0.016* |
| **Frontal_Inf_Oper_R2302** | 1.541 | 0.927 | 1.642 | | 0.223 | 9.146 | 0.007** |
| **Frontal_Inf_Tri_L2311** | 1.761 | 0.811 | 1.844 | | 0.214 | 11.358 | 0.003** |
| **Frontal_Inf_Tri_R2312** | 1.552 | 0.948 | 1.676 | | 0.275 | 7.105 | 0.016** |
| **Frontal_Inf_Orb_L2321** | 1.617 | 0.953 | 1.745 | | 0.185 | 12.978 | 0.002** |
| **Frontal_Inf_Orb_R2322** | 1.564 | 0.921 | 1.616 | | 0.263 | 6.763 | 0.018** |
| **Rolandic_Oper_L2331** | 1.595 | 1.045 | 1.621 | | 0.312 | 6.157 | 0.023** |
| **Rolandic_Oper_R2332** | 1.604 | 0.997 | 1.659 | | 0.259 | 8.273 | 0.01** |
| **Supp_Motor_Area_L2401** | 1.721 | 0.372 | 1.618 | | 0.282 | 0.606 | 0.446 |
| **Supp_Motor_Area_R2402** | 1.712 | 0.665 | 1.713 | | 0.285 | 4.206 | 0.055 |
| **OIfactory_L2501** | 1.569 | 1.027 | 1.453 | | 0.249 | 7.405 | 0.014** |
| **OIfactory_R2502** | 1.531 | 1.034 | 1.435 | | 0.202 | 9.307 | 0.007** |
| **Frontal_Sup_Medial_L2601** | 1.878 | 0.488 | 1.753 | | 0.258 | 1.403 | 0.252 |
| **Frontal_Sup_Medial_R2602** | 1.757 | 0.629 | 1.717 | | 0.273 | 3.005 | 0.1 |
| **Frontal_Mid_Orb_L2611** | 2.297 | 0.546 | 1.996 | | 0.356 | 0.302 | 0.589 |
| **Frontal_Mid_Orb_R2612** | 2.209 | 0.619 | 2.003 | | 0.356 | 0.879 | 0.361 |
| **Rectus_L2701** | 2.341 | 0.558 | 1.918 | | 0.355 | 1.598 | 0.222 |
| **Rectus_R2702** | 2.143 | 0.691 | 1.945 | | 0.362 | 1.038 | 0.322 |
| **Insula_L3001** | 1.585 | 1.027 | 1.675 | | 0.227 | 6.009 | 0.025* |
| **Insula_R3002** | 1.613 | 1.017 | 1.665 | | 0.233 | 6.565 | 0.02* |
| **Cingulum_Ant_L4001** | 2.07 | 0.655 | 1.887 | | 0.283 | 5.378 | 0.032* |
| **Cingulum_Ant_R4002** | 2.095 | 0.643 | 1.941 | | 0.304 | 2.92 | 0.105 |
| **Cingulum_Mid_L4011** | 2.35 | 0.539 | 2.08 | | 0.277 | 1.735 | 0.204 |
| **Cingulum_Mid_R4012** | 2.211 | 0.518 | 2.041 | | 0.26 | 4.766 | 0.043* |
| **Cingulum_Post_L4021** | 1.899 | 0.465 | 1.908 | | 0.204 | 5.202 | 0.035* |
| **Cingulum_Post_R4022** | 1.615 | 0.541 | 1.829 | | 0.233 | 12.645 | 0.002** |
| **Hippocampus_L4101** | 1.177 | 0.746 | 1.368 | | 0.193 | 6.811 | 0.018* |
| **Hippocampus_R4102** | 1.188 | 0.716 | 1.374 | | 0.224 | 5.402 | 0.032 |
| **ParaHippocampal_L4111** | 1.059 | 0.64 | 1.304 | | 0.146 | 7.801 | 0.012* |
| **ParaHippocampal_R4112** | 1.16 | 0.683 | 1.341 | | 0.175 | 7.674 | 0.013* |
| **Amygdala_L4201** | 1.253 | 0.149 | 1.253 | | 0.149 | 5.898 | 0.026* |
| **Amygdala_R4202** | 1.304 | 0.117 | 1.304 | | 0.117 | 8.045 | 0.011* |
| **Calcarine_L5001** | 1.537 | 0.577 | 1.507 | | 0.295 | 4.305 | 0.053 |
| **Calcarine_R5002** | 1.607 | 0.618 | 1.538 | | 0.309 | 4.302 | 0.053 |
| **Cuneus_L5011** | 1.32 | 0.774 | 1.525 | | 0.321 | 3.566 | 0.075 |
| **Cuneus_R5012** | 1.297 | 0.765 | 1.467 | | 0.31 | 3.596 | 0.074 |
| **Lingual_L5021** | 1.284 | 0.51 | 1.424 | | 0.22 | 3.217 | 0.09 |
| **Lingual_R5022** | 1.34 | 0.327 | 1.381 | | 0.222 | 0.284 | 0.601 |
| **Occipital_Sup_L5101** | 1.237 | 0.693 | 1.483 | | 0.306 | 3.466 | 0.079 |
| **Occipital_Sup_R5102** | 1.179 | 0.703 | 1.412 | | 0.294 | 4.034 | 0.06 |
| **Occipital_Mid_L5201** | 1.353 | 0.747 | 1.561 | | 0.299 | 5.219 | 0.035* |
| **Occipital_Mid_R5202** | 1.21 | 0.722 | 1.409 | | 0.298 | 4.339 | 0.052 |
| **Occipital_Inf_L5301** | 1.341 | 0.644 | 1.565 | | 0.315 | 3.014 | 0.1 |
| **Occipital_Inf_R5302** | 1.222 | 0.64 | 1.376 | | 0.275 | 4.609 | 0.046* |
| **Fusiform_L5401** | 1.273 | 0.697 | 1.441 | | 0.215 | 5.486 | 0.031* |
| **Fusiform_R5402** | 1.373 | 0.75 | 1.489 | | 0.22 | 7.87 | 0.012* |
| **Postcentral_L6001** | 1.295 | 0.816 | 1.546 | | 0.28 | 8.582 | 0.009** |
| **Postcentral_R6002** | 1.275 | 0.774 | 1.488 | | 0.257 | 6.195 | 0.023* |
| **Parietal_Sup_L6101** | 1.362 | 0.874 | 1.606 | | 0.321 | 10.699 | 0.004** |
| **Parietal_Sup_R6102** | 1.212 | 0.764 | 1.415 | | 0.302 | 6.111 | 0.024* |
| **Parietal_Inf_L6201** | 1.477 | 0.9 | 1.739 | | 0.308 | 7.338 | 0.014* |
| **Parietal_Inf_R6202** | 1.303 | 0.769 | 1.63 | | 0.313 | 6.353 | 0.021* |
| **SupraMarginal_L6211** | 1.336 | 0.773 | 1.708 | | 0.303 | 7.749 | 0.012* |
| **SupraMarginal_R6212** | 1.699 | 0.597 | 1.654 | | 0.274 | 7.17 | 0.015* |
| **Angular_L6221** | 1.683 | 0.569 | 1.782 | | 0.304 | 7.192 | 0.015* |
| **Angular_R6222** | 1.967 | 0.753 | 1.657 | | 0.275 | 8.818 | 0.008** |
| **Precuneus_L6301** | 1.968 | 0.726 | 1.85 | | 0.265 | 12.701 | 0.002** |
| **Precuneus_R6302** | 1.855 | 0.474 | 1.857 | | 0.259 | 7.961 | 0.011* |
| **Paracentral_Lobule_L6401** | 1.54 | 0.663 | 1.578 | | 0.264 | 8.201 | 0.01* |
| **Paracentral_Lobule_R6402** | 1.903 | 0.792 | 1.663 | | 0.261 | 6.614 | 0.019* |
| **Caudate_L7001** | 1.942 | 0.578 | 1.079 | | 0.204 | 5.74 | 0.028* |
| **Caudate_R7002** | 1.563 | 0.965 | 1.311 | | 0.261 | 15.35 | 0.001** |
| **Putamen_L7011** | 1.951 | 0.334 | 1.541 | | 0.927 | 9.678 | 0.006** |
| **Putamen_R7012** | 1.978 | 0.346 | 1.761 | | 0.811 | 6.796 | 0.018** |
| **Pallidum_L7021** | 1.552 | 0.948 | 1.954 | | 0.267 | 1.018 | 0.311 |
| **Pallidum_R7022** | 1.617 | 0.953 | 1.97 | | 0.26 | 13.214 | 0.002** |
| **Thalamus_L7101** | 1.564 | 0.921 | 1.45 | | 0.151 | 5.693 | 0.028** |
| **Thalamus_R7102** | 1.595 | 1.045 | 1.481 | | 0.181 | 9.805 | 0.006** |
| **Heschl_L8101** | 1.604 | 0.997 | 1.671 | | 0.323 | 7.62 | 0.013** |
| **Heschl_R8102** | 1.721 | 0.372 | 1.756 | | 0.294 | 10.057 | 0.005** |
| **Temporal_Sup_L8111** | 1.712 | 0.665 | 1.642 | | 0.301 | 6.437 | 0.021* |
| **Temporal_Sup_R8112** | 1.569 | 1.027 | 1.699 | | 0.286 | 6.18 | 0.023* |
| **Temporal_Pole_Sup_L8121** | 1.531 | 1.034 | 1.283 | | 0.22 | 6.239 | 0.022* |
| **Temporal_Pole_Sup_R8122** | 1.878 | 0.488 | 1.327 | | 0.227 | 6.522 | 0.02* |
| **Temporal_Mid_L8201** | 1.757 | 0.629 | 1.737 | | 0.301 | 2.677 | 0.119 |
| **Temporal_Mid_R8202** | 2.297 | 0.546 | 1.674 | | 0.272 | 5.707 | 0.028* |
| **Temporal_Pole_Mid_L8211** | 2.209 | 0.619 | 1.443 | | 0.239 | 4.655 | 0.045* |
| **Temporal_Pole_Mid_R8212** | 2.341 | 0.558 | 1.452 | | 0.239 | 5.204 | 0.035* |
| **Temporal_Inf_L8301** | 2.143 | 0.691 | 1.447 | | 0.32 | 2.025 | 0.172 |
| **Temporal_Inf_R8302** | 1.585 | 1.027 | 1.461 | | 0.28 | 3.093 | 0.096 |

**Supplementary Table 4. SUVR between EOFAD and NC groups on entire 90 ROIs.**

|  | **EOFAD** | | **NC** | | **T** | **P** |
| --- | --- | --- | --- | --- | --- | --- |
|  | **Mean** | **SD** | **Mean** | **SD** |  |  |
| **Precentral_L2001** | 1.303 | 0.769 | 1.309 | 0.139 | 10.59 | 0.006** |
| **Precentral_R2002** | 1.336 | 0.773 | 1.261 | 0.100 | 10.295 | 0.006** |
| **Frontal_Sup_L2101** | 1.699 | 0.597 | 1.298 | 0.193 | 9.737 | 0.008** |
| **Frontal_Sup_R2102** | 1.683 | 0.569 | 1.231 | 0.203 | 5.01 | 0.042* |
| **Frontal_Sup_Orb_L2111** | 1.967 | 0.753 | 1.484 | 0.207 | 7.732 | 0.015* |
| **Frontal_Sup_Orb_R2112** | 1.968 | 0.726 | 1.418 | 0.232 | 5.546 | 0.034* |
| **Frontal_Mid_L2201** | 1.855 | 0.474 | 1.317 | 0.215 | 6.216 | 0.026* |
| **Frontal_Mid_R2202** | 1.540 | 0.663 | 1.142 | 0.200 | 7.299 | 0.017* |
| **Frontal_Mid_Orb_L2211** | 1.903 | 0.792 | 1.462 | 0.216 | 13.898 | 0.002** |
| **Frontal_Mid_Orb_R2212** | 1.942 | 0.578 | 1.318 | 0.244 | 3.611 | 0.078 |
| **Frontal_Inf_Oper_L2301** | 1.563 | 0.965 | 1.337 | 0.176 | 7.508 | 0.016* |
| **Frontal_Inf_Oper_R2302** | 1.541 | 0.927 | 1.324 | 0.161 | 9.029 | 0.009** |
| **Frontal_Inf_Tri_L2311** | 1.761 | 0.811 | 1.405 | 0.176 | 10.655 | 0.006** |
| **Frontal_Inf_Tri_R2312** | 1.552 | 0.948 | 1.268 | 0.174 | 8.943 | 0.01* |
| **Frontal_Inf_Orb_L2321** | 1.617 | 0.953 | 1.281 | 0.160 | 10.738 | 0.006** |
| **Frontal_Inf_Orb_R2322** | 1.564 | 0.921 | 1.212 | 0.197 | 7.556 | 0.016* |
| **Rolandic_Oper_L2331** | 1.595 | 1.045 | 1.244 | 0.191 | 7.783 | 0.014* |
| **Rolandic_Oper_R2332** | 1.604 | 0.997 | 1.319 | 0.112 | 10.779 | 0.005** |
| **Supp_Motor_Area_L2401** | 1.721 | 0.372 | 1.124 | 0.163 | 5.791 | 0.03* |
| **Supp_Motor_Area_R2402** | 1.712 | 0.665 | 1.292 | 0.166 | 8.532 | 0.011* |
| **OIfactory_L2501** | 1.569 | 1.027 | 1.123 | 0.149 | 8.351 | 0.012* |
| **OIfactory_R2502** | 1.531 | 1.034 | 1.139 | 0.150 | 8.501 | 0.011* |
| **Frontal_Sup_Medial_L2601** | 1.878 | 0.488 | 1.155 | 0.218 | 2.708 | 0.122 |
| **Frontal_Sup_Medial_R2602** | 1.757 | 0.629 | 1.177 | 0.218 | 4.706 | 0.048* |
| **Frontal_Mid_Orb_L2611** | 2.297 | 0.546 | 1.285 | 0.276 | 1.718 | 0.211 |
| **Frontal_Mid_Orb_R2612** | 2.209 | 0.619 | 1.401 | 0.268 | 2.944 | 0.108 |
| **Rectus_L2701** | 2.341 | 0.558 | 1.258 | 0.269 | 4.703 | 0.048* |
| **Rectus_R2702** | 2.143 | 0.691 | 1.349 | 0.275 | 2.882 | 0.112 |
| **Insula_L3001** | 1.585 | 1.027 | 1.292 | 0.116 | 6.498 | 0.023* |
| **Insula_R3002** | 1.613 | 1.017 | 1.319 | 0.132 | 7.346 | 0.017* |
| **Cingulum_Ant_L4001** | 2.070 | 0.655 | 1.366 | 0.091 | 15.679 | 0.001** |
| **Cingulum_Ant_R4002** | 2.095 | 0.643 | 1.470 | 0.113 | 10.123 | 0.007** |
| **Cingulum_Mid_L4011** | 2.350 | 0.539 | 1.430 | 0.158 | 5.288 | 0.037* |
| **Cingulum_Mid_R4012** | 2.211 | 0.518 | 1.541 | 0.151 | 12.066 | 0.004** |
| **Cingulum_Post_L4021** | 1.899 | 0.465 | 1.503 | 0.167 | 6.605 | 0.022* |
| **Cingulum_Post_R4022** | 1.615 | 0.541 | 1.574 | 0.160 | 20.534 | 0.000*** |
| **Hippocampus_L4101** | 1.177 | 0.746 | 1.349 | 0.105 | 7.949 | 0.014* |
| **Hippocampus_R4102** | 1.188 | 0.716 | 1.431 | 0.091 | 8.793 | 0.01* |
| **ParaHippocampal_L4111** | 1.059 | 0.640 | 1.203 | 0.118 | 6.664 | 0.022* |
| **ParaHippocampal_R4112** | 1.160 | 0.683 | 1.279 | 0.113 | 8.052 | 0.013* |
| **Amygdala_L4201** | 1.422 | 0.209 | 1.253 | 0.149 | 6.243 | 0.026* |
| **Amygdala_R4202** | 1.567 | 0.176 | 1.304 | 0.117 | 7.854 | 0.014* |
| **Calcarine_L5001** | 1.537 | 0.577 | 1.290 | 0.203 | 8.76 | 0.01* |
| **Calcarine_R5002** | 1.607 | 0.618 | 1.397 | 0.191 | 9.808 | 0.007* |
| **Cuneus_L5011** | 1.320 | 0.774 | 1.367 | 0.184 | 6.565 | 0.023* |
| **Cuneus_R5012** | 1.297 | 0.765 | 1.350 | 0.192 | 5.73 | 0.031* |
| **Lingual_L5021** | 1.284 | 0.510 | 1.259 | 0.180 | 4.286 | 0.057 |
| **Lingual_R5022** | 1.340 | 0.327 | 1.254 | 0.142 | 1.985 | 0.181 |
| **Occipital_Sup_L5101** | 1.237 | 0.693 | 1.336 | 0.176 | 6.61 | 0.022* |
| **Occipital_Sup_R5102** | 1.179 | 0.703 | 1.292 | 0.193 | 6.271 | 0.025* |
| **Occipital_Mid_L5201** | 1.353 | 0.747 | 1.293 | 0.220 | 7.041 | 0.019* |
| **Occipital_Mid_R5202** | 1.210 | 0.722 | 1.208 | 0.216 | 6.092 | 0.027* |
| **Occipital_Inf_L5301** | 1.341 | 0.644 | 1.290 | 0.255 | 4.442 | 0.054 |
| **Occipital_Inf_R5302** | 1.222 | 0.640 | 1.234 | 0.197 | 6.837 | 0.02* |
| **Fusiform_L5401** | 1.273 | 0.697 | 1.205 | 0.170 | 5.836 | 0.03* |
| **Fusiform_R5402** | 1.373 | 0.750 | 1.313 | 0.170 | 8.03 | 0.013* |
| **Postcentral_L6001** | 1.295 | 0.816 | 1.196 | 0.164 | 12.915 | 0.003** |
| **Postcentral_R6002** | 1.275 | 0.774 | 1.189 | 0.165 | 8.117 | 0.013* |
| **Parietal_Sup_L6101** | 1.362 | 0.874 | 1.195 | 0.246 | 13.547 | 0.002** |
| **Parietal_Sup_R6102** | 1.212 | 0.764 | 1.063 | 0.240 | 7.431 | 0.016* |
| **Parietal_Inf_L6201** | 1.477 | 0.900 | 1.248 | 0.246 | 8.064 | 0.013* |
| **Parietal_Inf_R6202** | 1.303 | 0.769 | 1.168 | 0.247 | 7.581 | 0.016* |
| **SupraMarginal_L6211** | 1.336 | 0.773 | 1.251 | 0.235 | 8.552 | 0.011* |
| **SupraMarginal_R6212** | 1.699 | 0.597 | 1.249 | 0.142 | 10.884 | 0.005** |
| **Angular_L6221** | 1.683 | 0.569 | 1.346 | 0.270 | 6.704 | 0.021* |
| **Angular_R6222** | 1.967 | 0.753 | 1.298 | 0.235 | 8.423 | 0.012* |
| **Precuneus_L6301** | 1.968 | 0.726 | 1.310 | 0.264 | 10.467 | 0.006 |
| **Precuneus_R6302** | 1.855 | 0.474 | 1.371 | 0.286 | 5.148 | 0.04* |
| **Paracentral_Lobule_L6401** | 1.540 | 0.663 | 1.229 | 0.173 | 10.592 | 0.006** |
| **Paracentral_Lobule_R6402** | 1.903 | 0.792 | 1.276 | 0.218 | 6.471 | 0.023* |
| **Caudate_L7001** | 1.942 | 0.578 | 0.852 | 0.291 | 1.135 | 0.305 |
| **Caudate_R7002** | 1.563 | 0.965 | 1.073 | 0.328 | 7.77 | 0.015* |
| **Putamen_L7011** | 1.951 | 0.334 | 1.502 | 0.112 | 7.269 | 0.017* |
| **Putamen_R7012** | 1.978 | 0.346 | 1.547 | 0.133 | 31.15 | 0.000*** |
| **Pallidum_L7021** | 1.552 | 0.948 | 1.750 | 0.138 | 18.246 | 0.001* |
| **Pallidum_R7022** | 1.617 | 0.953 | 1.762 | 0.124 | 15.606 | 0.001* |
| **Thalamus_L7101** | 1.564 | 0.921 | 1.298 | 0.151 | 4.326 | 0.056 |
| **Thalamus_R7102** | 1.595 | 1.045 | 1.376 | 0.148 | 9.18 | 0.009* |
| **Heschl_L8101** | 1.604 | 0.997 | 1.363 | 0.160 | 10.38 | 0.006** |
| **Heschl_R8102** | 1.721 | 0.372 | 1.471 | 0.107 | 14.219 | 0.002** |
| **Temporal_Sup_L8111** | 1.712 | 0.665 | 1.280 | 0.190 | 8.645 | 0.011* |
| **Temporal_Sup_R8112** | 1.569 | 1.027 | 1.347 | 0.168 | 8.379 | 0.012* |
| **Temporal_Pole_Sup_L8121** | 1.531 | 1.034 | 1.080 | 0.133 | 7.542 | 0.016* |
| **Temporal_Pole_Sup_R8122** | 1.878 | 0.488 | 1.132 | 0.157 | 7.753 | 0.015* |
| **Temporal_Mid_L8201** | 1.757 | 0.629 | 1.355 | 0.233 | 4.758 | 0.047* |
| **Temporal_Mid_R8202** | 2.297 | 0.546 | 1.339 | 0.211 | 6.61 | 0.022* |
| **Temporal_Pole_Mid_L8211** | 2.209 | 0.619 | 1.199 | 0.156 | 6.43 | 0.024* |
| **Temporal_Pole_Mid_R8212** | 2.341 | 0.558 | 1.212 | 0.150 | 7.681 | 0.015* |
| **Temporal_Inf_L8301** | 2.143 | 0.691 | 1.117 | 0.218 | 4.44 | 0.054 |
| **Temporal_Inf_R8302** | 1.585 | 1.027 | 1.219 | 0.197 | 4.753 | 0.047* |

**Supplementary Table 5. Correlations between striatum and cortex in EOFAD**

|  | | **Amygdala_L4201** | **Amygdala_R4202** | **Caudate_L7001** | **Caudate_R7002** | **Putamen_L7011** | **Putamen_R7012** |
| --- | --- | --- | --- | --- | --- | --- | --- |
| **Precentral_L2001** | **R** | .913* | .960** | 0.825 | 0.743 | -0.196 | 0.621 |
|  | **P** | 0.030 | 0.009 | 0.086 | 0.150 | 0.752 | 0.264 |
| **Precentral_R2002** | **R** | .888* | .942* | 0.875 | 0.797 | -0.183 | 0.639 |
|  | **P** | 0.044 | 0.017 | 0.052 | 0.106 | 0.768 | 0.246 |
| **Frontal_Sup_L2101** | **R** | .914* | .938* | 0.764 | 0.680 | -0.116 | 0.624 |
|  | **P** | 0.030 | 0.018 | 0.133 | 0.207 | 0.853 | 0.260 |
| **Frontal_Sup_R2102** | **R** | .954* | .979** | 0.855 | 0.792 | -0.074 | 0.709 |
|  | **P** | 0.012 | 0.004 | 0.065 | 0.111 | 0.905 | 0.180 |
| **Frontal_Sup_Orb_L2111** | **R** | .945* | .948* | 0.716 | 0.657 | -0.040 | 0.661 |
|  | **P** | 0.016 | 0.014 | 0.174 | 0.229 | 0.950 | 0.224 |
| **Frontal_Sup_Orb_R2112** | **R** | .956* | .949* | 0.733 | 0.685 | 0.026 | 0.705 |
|  | **P** | 0.011 | 0.014 | 0.158 | 0.202 | 0.967 | 0.183 |
| **Frontal_Mid_L2201** | **R** | 0.810 | 0.791 | 0.641 | 0.560 | 0.079 | 0.602 |
|  | **P** | 0.097 | 0.111 | 0.244 | 0.326 | 0.900 | 0.283 |
| **Frontal_Mid_R2202** | **R** | .941* | .966** | 0.866 | 0.797 | -0.060 | 0.709 |
|  | **P** | 0.017 | 0.008 | 0.058 | 0.107 | 0.923 | 0.180 |
| **Frontal_Mid_Orb_L2211** | **R** | .882* | .902* | 0.676 | 0.587 | -0.138 | 0.562 |
|  | **P** | 0.048 | 0.036 | 0.210 | 0.298 | 0.825 | 0.325 |
| **Frontal_Mid_Orb_R2212** | **R** | 0.871 | 0.835 | 0.591 | 0.548 | 0.130 | 0.651 |
|  | **P** | 0.054 | 0.079 | 0.294 | 0.339 | 0.834 | 0.234 |
| **Frontal_Inf_Oper_L2301** | **R** | .973** | .989** | 0.806 | 0.754 | -0.061 | 0.708 |
|  | **P** | 0.005 | 0.001 | 0.100 | 0.141 | 0.923 | 0.181 |
| **Frontal_Inf_Oper_R2302** | **R** | .952* | .978** | 0.860 | 0.797 | -0.075 | 0.710 |
|  | **P** | 0.013 | 0.004 | 0.061 | 0.106 | 0.904 | 0.179 |
| **Frontal_Inf_Tri_L2311** | **R** | .949* | .959* | 0.781 | 0.716 | -0.035 | 0.689 |
|  | **P** | 0.014 | 0.010 | 0.119 | 0.173 | 0.955 | 0.198 |
| **Frontal_Inf_Tri_R2312** | **R** | .964** | .984** | 0.840 | 0.782 | -0.057 | 0.716 |
|  | **P** | 0.008 | 0.003 | 0.075 | 0.118 | 0.927 | 0.174 |
| **Frontal_Inf_Orb_L2321** | **R** | .950* | .965** | 0.739 | 0.676 | -0.090 | 0.652 |
|  | **P** | 0.013 | 0.008 | 0.154 | 0.210 | 0.885 | 0.233 |
| **Frontal_Inf_Orb_R2322** | **R** | .974** | .985** | 0.819 | 0.767 | -0.025 | 0.728 |
|  | **P** | 0.005 | 0.002 | 0.090 | 0.130 | 0.968 | 0.163 |
| **Rolandic_Oper_L2331** | **R** | .972** | .964** | .882* | 0.841 | 0.116 | 0.814 |
|  | **P** | 0.006 | 0.008 | 0.048 | 0.074 | 0.852 | 0.093 |
| **Rolandic_Oper_R2332** | **R** | .945* | .958* | .911* | 0.854 | 0.029 | 0.772 |
|  | **P** | 0.015 | 0.010 | 0.031 | 0.065 | 0.962 | 0.126 |
| **Supp_Motor_Area_L2401** | **R** | 0.782 | 0.763 | 0.542 | 0.463 | 0.036 | 0.533 |
|  | **P** | 0.118 | 0.134 | 0.346 | 0.433 | 0.954 | 0.355 |
| **Supp_Motor_Area_R2402** | **R** | .952* | .972** | 0.842 | 0.776 | -0.057 | 0.707 |
|  | **P** | 0.013 | 0.006 | 0.073 | 0.123 | 0.927 | 0.181 |
| **OIfactory_L2501** | **R** | .986** | .951* | 0.840 | 0.833 | 0.239 | 0.870 |
|  | **P** | 0.002 | 0.013 | 0.075 | 0.080 | 0.699 | 0.055 |
| **OIfactory_R2502** | **R** | .991** | .964** | 0.869 | 0.859 | 0.209 | 0.871 |
|  | **P** | 0.001 | 0.008 | 0.056 | 0.062 | 0.736 | 0.055 |
| **Frontal_Sup_Medial_L2601** | **R** | .901* | 0.857 | 0.680 | 0.646 | 0.213 | 0.738 |
|  | **P** | 0.037 | 0.063 | 0.207 | 0.239 | 0.731 | 0.154 |
| **Frontal_Sup_Medial_R2602** | **R** | .974** | .970** | 0.852 | 0.807 | 0.072 | 0.782 |
|  | **P** | 0.005 | 0.006 | 0.067 | 0.099 | 0.909 | 0.118 |
| **Frontal_Mid_Orb_L2611** | **R** | 0.452 | 0.337 | 0.187 | 0.196 | 0.507 | 0.472 |
|  | **P** | 0.444 | 0.579 | 0.764 | 0.752 | 0.383 | 0.422 |
| **Frontal_Mid_Orb_R2612** | **R** | 0.859 | 0.783 | 0.609 | 0.610 | 0.371 | 0.771 |
|  | **P** | 0.062 | 0.117 | 0.276 | 0.275 | 0.539 | 0.127 |
| **Rectus_L2701** | **R** | 0.230 | 0.108 | -0.063 | -0.042 | 0.496 | 0.273 |
|  | **P** | 0.709 | 0.863 | 0.920 | 0.947 | 0.396 | 0.657 |
| **Rectus_R2702** | **R** | .911* | 0.863 | 0.661 | 0.638 | 0.222 | 0.744 |
|  | **P** | 0.031 | 0.059 | 0.225 | 0.247 | 0.720 | 0.149 |
| **Insula_L3001** | **R** | .993** | .981** | 0.843 | 0.817 | 0.109 | 0.813 |
|  | **P** | 0.001 | 0.003 | 0.073 | 0.091 | 0.861 | 0.094 |
| **Insula_R3002** | **R** | .982** | .981** | .882* | 0.845 | 0.079 | 0.807 |
|  | **P** | 0.003 | 0.003 | 0.048 | 0.071 | 0.899 | 0.099 |
| **Cingulum_Ant_L4001** | **R** | .935* | 0.875 | 0.820 | 0.819 | 0.383 | .898* |
|  | **P** | 0.020 | 0.052 | 0.089 | 0.090 | 0.525 | 0.039 |
| **Cingulum_Ant_R4002** | **R** | .948* | .884* | 0.830 | 0.851 | 0.416 | .934* |
|  | **P** | 0.014 | 0.047 | 0.082 | 0.067 | 0.486 | 0.020 |
| **Cingulum_Mid_L4011** | **R** | 0.344 | 0.251 | 0.235 | 0.194 | 0.445 | 0.391 |
|  | **P** | 0.571 | 0.684 | 0.703 | 0.754 | 0.453 | 0.515 |
| **Cingulum_Mid_R4012** | **R** | 0.823 | 0.764 | 0.702 | 0.664 | 0.340 | 0.760 |
|  | **P** | 0.087 | 0.133 | 0.186 | 0.221 | 0.575 | 0.136 |
| **Cingulum_Post_L4021** | **R** | 0.549 | 0.507 | 0.394 | 0.306 | 0.162 | 0.411 |
|  | **P** | 0.338 | 0.383 | 0.512 | 0.617 | 0.795 | 0.492 |
| **Cingulum_Post_R4022** | **R** | 0.819 | 0.860 | 0.762 | 0.647 | -0.177 | 0.540 |
|  | **P** | 0.090 | 0.061 | 0.134 | 0.238 | 0.776 | 0.347 |
| **Hippocampus_L4101** | **R** | .979** | .980** | 0.866 | 0.823 | 0.058 | 0.787 |
|  | **P** | 0.004 | 0.003 | 0.058 | 0.087 | 0.927 | 0.114 |
| **Hippocampus_R4102** | **R** | .967** | .987** | 0.858 | 0.804 | -0.049 | 0.731 |
|  | **P** | 0.007 | 0.002 | 0.063 | 0.101 | 0.937 | 0.161 |
| **ParaHippocampal_L4111** | **R** | .970** | .992** | 0.813 | 0.760 | -0.088 | 0.699 |
|  | **P** | 0.006 | 0.001 | 0.094 | 0.136 | 0.889 | 0.189 |
| **ParaHippocampal_R4112** | **R** | .953* | .988** | 0.810 | 0.750 | -0.151 | 0.662 |
|  | **P** | 0.012 | 0.002 | 0.096 | 0.145 | 0.808 | 0.224 |
| **Amygdala_L4201** | **R** | 1.000 | .985** | 0.828 | 0.820 | 0.123 | 0.826 |
|  | **P** | 0.000 | 0.002 | 0.084 | 0.089 | 0.844 | 0.085 |
| **Amygdala_R4202** | **R** | .985** | 1.000 | 0.823 | 0.792 | -0.039 | 0.742 |
|  | **P** | 0.002 | 0.000 | 0.087 | 0.111 | 0.950 | 0.151 |
| **Calcarine_L5001** | **R** | .918* | 0.869 | 0.834 | 0.810 | 0.330 | 0.862 |
|  | **P** | 0.028 | 0.056 | 0.079 | 0.096 | 0.587 | 0.060 |
| **Calcarine_R5002** | **R** | .911* | .894* | .903* | 0.852 | 0.197 | 0.821 |
|  | **P** | 0.032 | 0.041 | 0.036 | 0.067 | 0.750 | 0.089 |
| **Cuneus_L5011** | **R** | .902* | .946* | .898* | 0.825 | -0.122 | 0.681 |
|  | **P** | 0.036 | 0.015 | 0.038 | 0.085 | 0.846 | 0.205 |
| **Cuneus_R5012** | **R** | .932* | .959** | .915* | 0.854 | -0.033 | 0.742 |
|  | **P** | 0.021 | 0.010 | 0.029 | 0.065 | 0.958 | 0.151 |
| **Lingual_L5021** | **R** | .966** | .988** | .882* | 0.853 | -0.031 | 0.763 |
|  | **P** | 0.008 | 0.002 | 0.048 | 0.066 | 0.961 | 0.133 |
| **Lingual_R5022** | **R** | .970** | .984** | .910* | 0.877 | 0.029 | 0.797 |
|  | **P** | 0.006 | 0.002 | 0.032 | 0.051 | 0.964 | 0.106 |
| **Occipital_Sup_L5101** | **R** | .908* | .958* | 0.852 | 0.774 | -0.186 | 0.637 |
|  | **P** | 0.033 | 0.010 | 0.067 | 0.124 | 0.765 | 0.248 |
| **Occipital_Sup_R5102** | **R** | .931* | .964** | .889* | 0.820 | -0.080 | 0.708 |
|  | **P** | 0.021 | 0.008 | 0.044 | 0.089 | 0.898 | 0.181 |
| **Occipital_Mid_L5201** | **R** | .947* | .970** | 0.842 | 0.773 | -0.071 | 0.698 |
|  | **P** | 0.015 | 0.006 | 0.074 | 0.125 | 0.909 | 0.190 |
| **Occipital_Mid_R5202** | **R** | .923* | .953* | .899* | 0.829 | -0.059 | 0.716 |
|  | **P** | 0.025 | 0.012 | 0.038 | 0.083 | 0.925 | 0.174 |
| **Occipital_Inf_L5301** | **R** | .956* | .968** | 0.833 | 0.770 | -0.018 | 0.722 |
|  | **P** | 0.011 | 0.007 | 0.080 | 0.128 | 0.977 | 0.168 |
| **Occipital_Inf_R5302** | **R** | .919* | .950* | .879* | 0.802 | -0.082 | 0.693 |
|  | **P** | 0.027 | 0.013 | 0.050 | 0.102 | 0.896 | 0.195 |
| **Fusiform_L5401** | **R** | .982** | .996** | 0.869 | 0.835 | -0.006 | 0.772 |
|  | **P** | 0.003 | 0.000 | 0.056 | 0.078 | 0.993 | 0.126 |
| **Fusiform_R5402** | **R** | .925* | .974** | 0.844 | 0.786 | -0.185 | 0.654 |
|  | **P** | 0.024 | 0.005 | 0.073 | 0.115 | 0.765 | 0.232 |
| **Postcentral_L6001** | **R** | .933* | .955* | 0.843 | 0.768 | -0.060 | 0.693 |
|  | **P** | 0.021 | 0.011 | 0.073 | 0.129 | 0.924 | 0.194 |
| **Postcentral_R6002** | **R** | 0.862 | .905* | .921* | 0.841 | -0.087 | 0.682 |
|  | **P** | 0.060 | 0.035 | 0.026 | 0.074 | 0.890 | 0.204 |
| **Parietal_Sup_L6101** | **R** | .886* | .920* | 0.812 | 0.717 | -0.125 | 0.622 |
|  | **P** | 0.045 | 0.027 | 0.095 | 0.173 | 0.841 | 0.262 |
| **Parietal_Sup_R6102** | **R** | .887* | .914* | .909* | 0.829 | -0.024 | 0.711 |
|  | **P** | 0.045 | 0.030 | 0.033 | 0.083 | 0.969 | 0.178 |
| **Parietal_Inf_L6201** | **R** | .939* | .970** | 0.818 | 0.744 | -0.128 | 0.660 |
|  | **P** | 0.018 | 0.006 | 0.091 | 0.149 | 0.838 | 0.226 |
| **Parietal_Inf_R6202** | **R** | .902* | .938* | .892* | 0.812 | -0.092 | 0.685 |
|  | **P** | 0.036 | 0.018 | 0.042 | 0.095 | 0.883 | 0.202 |
| **SupraMarginal_L6211** | **R** | .956* | .968** | 0.813 | 0.749 | -0.034 | 0.707 |
|  | **P** | 0.011 | 0.007 | 0.094 | 0.145 | 0.957 | 0.182 |
| **SupraMarginal_R6212** | **R** | .926* | .959** | .879* | 0.806 | -0.088 | 0.696 |
|  | **P** | 0.024 | 0.010 | 0.050 | 0.100 | 0.888 | 0.192 |
| **Angular_L6221** | **R** | .947* | .977** | 0.792 | 0.723 | -0.134 | 0.653 |
|  | **P** | 0.014 | 0.004 | 0.110 | 0.168 | 0.830 | 0.232 |
| **Angular_R6222** | **R** | .930* | .955* | .879* | 0.807 | -0.049 | 0.713 |
|  | **P** | 0.022 | 0.011 | 0.050 | 0.099 | 0.937 | 0.177 |
| **Precuneus_L6301** | **R** | 0.859 | 0.857 | 0.733 | 0.647 | 0.020 | 0.637 |
|  | **P** | 0.062 | 0.063 | 0.159 | 0.238 | 0.974 | 0.248 |
| **Precuneus_R6302** | **R** | .888* | 0.865 | 0.751 | 0.689 | 0.144 | 0.720 |
|  | **P** | 0.044 | 0.058 | 0.143 | 0.198 | 0.817 | 0.171 |
| **Paracentral_Lobule_L6401** | **R** | .900* | .948* | 0.834 | 0.747 | -0.191 | 0.618 |
|  | **P** | 0.037 | 0.014 | 0.079 | 0.147 | 0.758 | 0.267 |
| **Paracentral_Lobule_R6402** | **R** | .926* | .965** | .881* | 0.812 | -0.118 | 0.688 |
|  | **P** | 0.024 | 0.008 | 0.048 | 0.095 | 0.850 | 0.199 |
| **Caudate_L7001** | **R** | 0.828 | 0.823 | 1.000** | .981** | 0.252 | 0.867 |
|  | **P** | 0.084 | 0.087 | 0.000 | 0.003 | 0.683 | 0.057 |
| **Caudate_R7002** | **R** | 0.820 | 0.792 | .981** | 1.000** | 0.384 | .929* |
|  | **P** | 0.089 | 0.111 | 0.003 | 0.000 | 0.523 | 0.022 |
| **Putamen_L7011** | **R** | 0.123 | -0.039 | 0.252 | 0.384 | 1.000** | 0.621 |
|  | **P** | 0.844 | 0.950 | 0.683 | 0.523 | 0.000 | 0.264 |
| **Putamen_R7012** | **R** | 0.826 | 0.742 | 0.867 | .929* | 0.621 | 1.000** |
|  | **P** | 0.085 | 0.151 | 0.057 | 0.022 | 0.264 | 0.000 |
| **Pallidum_L7021** | **R** | .947* | .930* | .946* | .956* | 0.242 | .910* |
|  | **P** | 0.014 | 0.022 | 0.015 | 0.011 | 0.695 | 0.032 |
| **Pallidum_R7022** | **R** | .947* | .954* | .925* | .921* | 0.094 | 0.836 |
|  | **P** | 0.015 | 0.012 | 0.024 | 0.026 | 0.880 | 0.077 |
| **Thalamus_L7101** | **R** | 0.757 | 0.811 | .941* | 0.876 | -0.079 | 0.658 |
|  | **P** | 0.138 | 0.096 | 0.017 | 0.051 | 0.900 | 0.227 |
| **Thalamus_R7102** | **R** | 0.846 | 0.874 | .971** | .940* | 0.047 | 0.777 |
|  | **P** | 0.071 | 0.052 | 0.006 | 0.018 | 0.941 | 0.122 |
| **Heschl_L8101** | **R** | .972** | .951* | 0.873 | 0.843 | 0.188 | 0.845 |
|  | **P** | 0.006 | 0.013 | 0.053 | 0.073 | 0.762 | 0.072 |
| **Heschl_R8102** | **R** | .949* | .938* | .930* | .892* | 0.173 | 0.848 |
|  | **P** | 0.014 | 0.018 | 0.022 | 0.042 | 0.781 | 0.070 |
| **Temporal_Sup_L8111** | **R** | .966** | .934* | 0.826 | 0.800 | 0.216 | 0.835 |
|  | **P** | 0.007 | 0.020 | 0.085 | 0.104 | 0.727 | 0.079 |
| **Temporal_Sup_R8112** | **R** | .957* | .966** | .908* | 0.857 | 0.048 | 0.787 |
|  | **P** | 0.011 | 0.007 | 0.033 | 0.063 | 0.940 | 0.114 |
| **Temporal_Pole_Sup_L8121** | **R** | .952* | .951* | 0.647 | 0.613 | -0.051 | 0.646 |
|  | **P** | 0.012 | 0.013 | 0.238 | 0.272 | 0.935 | 0.239 |
| **Temporal_Pole_Sup_R8122** | **R** | .967** | .983** | 0.850 | 0.794 | -0.030 | 0.734 |
|  | **P** | 0.007 | 0.003 | 0.068 | 0.108 | 0.961 | 0.158 |
| **Temporal_Mid_L8201** | **R** | .892* | 0.862 | 0.710 | 0.657 | 0.156 | 0.714 |
|  | **P** | 0.042 | 0.060 | 0.179 | 0.228 | 0.803 | 0.176 |
| **Temporal_Mid_R8202** | **R** | .947* | .949* | .891* | 0.834 | 0.073 | 0.781 |
|  | **P** | 0.015 | 0.014 | 0.042 | 0.079 | 0.908 | 0.119 |
| **Temporal_Pole_Mid_L8211** | **R** | .920* | .960** | 0.674 | 0.605 | -0.249 | 0.548 |
|  | **P** | 0.027 | 0.009 | 0.212 | 0.279 | 0.686 | 0.339 |
| **Temporal_Pole_Mid_R8212** | **R** | .909* | .966** | 0.773 | 0.698 | -0.271 | 0.573 |
|  | **P** | 0.032 | 0.007 | 0.126 | 0.190 | 0.659 | 0.313 |
| **Temporal_Inf_L8301** | **R** | .970** | .982** | 0.794 | 0.740 | -0.049 | 0.705 |
|  | **P** | 0.006 | 0.003 | 0.109 | 0.153 | 0.937 | 0.184 |
| **Temporal_Inf_R8302** | **R** | .947* | .980** | 0.858 | 0.795 | -0.110 | 0.693 |
|  | **P** | 0.015 | 0.003 | 0.063 | 0.108 | 0.860 | 0.194 |

**Supplementary Table 6. Correlations between PiB accumulations and neuropsychological test in EOFAD**

|  | | **MMSE** | **MOCA** | **CDT** | **BNT** | **TMTA** | **TMTB** | **CDR** | **NPI** |
| --- | --- | --- | --- | --- | --- | --- | --- | --- | --- |
| **Precentral_L2001** | **R** | -0.045 | -0.338 | 0.068 | -0.282 | -0.560 | -0.089 | -0.318 | -0.143 |
|  | **P** | 0.943 | 0.578 | 0.913 | 0.646 | 0.326 | 0.887 | 0.602 | 0.818 |
| **Precentral_R2002** | **R** | -0.031 | -0.313 | 0.085 | -0.250 | -0.532 | -0.024 | -0.299 | -0.075 |
|  | **P** | 0.960 | 0.608 | 0.891 | 0.684 | 0.356 | 0.970 | 0.625 | 0.905 |
| **Frontal_Sup_L2101** | **R** | -0.073 | -0.387 | 0.060 | -0.316 | -0.500 | -0.095 | -0.298 | -0.187 |
|  | **P** | 0.907 | 0.520 | 0.924 | 0.604 | 0.391 | 0.879 | 0.626 | 0.763 |
| **Frontal_Sup_R2102** | **R** | 0.053 | -0.258 | 0.179 | -0.186 | -0.582 | -0.133 | -0.400 | -0.222 |
|  | **P** | 0.933 | 0.675 | 0.774 | 0.764 | 0.303 | 0.831 | 0.505 | 0.719 |
| **Frontal_Sup_Orb_L2111** | **R** | 0.054 | -0.264 | 0.173 | -0.200 | -0.589 | -0.248 | -0.427 | -0.352 |
|  | **P** | 0.931 | 0.668 | 0.781 | 0.747 | 0.296 | 0.688 | 0.473 | 0.561 |
| **Frontal_Sup_Orb_R2112** | **R** | 0.107 | -0.217 | 0.232 | -0.145 | -0.593 | -0.260 | -0.465 | -0.382 |
|  | **P** | 0.864 | 0.726 | 0.707 | 0.816 | 0.292 | 0.672 | 0.430 | 0.526 |
| **Frontal_Mid_L2201** | **R** | -0.123 | -0.440 | 0.050 | -0.334 | -0.284 | -0.003 | -0.196 | -0.165 |
|  | **P** | 0.844 | 0.458 | 0.936 | 0.582 | 0.644 | 0.996 | 0.752 | 0.791 |
| **Frontal_Mid_R2202** | **R** | 0.018 | -0.296 | 0.156 | -0.215 | -0.532 | -0.076 | -0.356 | -0.174 |
|  | **P** | 0.978 | 0.628 | 0.803 | 0.728 | 0.356 | 0.903 | 0.556 | 0.779 |
| **Frontal_Mid_Orb_L2211** | **R** | -0.117 | -0.425 | 0.010 | -0.362 | -0.477 | -0.122 | -0.268 | -0.211 |
|  | **P** | 0.852 | 0.476 | 0.987 | 0.549 | 0.417 | 0.845 | 0.663 | 0.734 |
| **Frontal_Mid_Orb_R2212** | **R** | 0.087 | -0.230 | 0.222 | -0.148 | -0.480 | -0.264 | -0.419 | -0.419 |
|  | **P** | 0.889 | 0.710 | 0.720 | 0.812 | 0.413 | 0.668 | 0.482 | 0.482 |
| **Frontal_Inf_Oper_L2301** | **R** | 0.110 | -0.203 | 0.222 | -0.142 | -0.647 | -0.241 | -0.471 | -0.329 |
|  | **P** | 0.860 | 0.743 | 0.720 | 0.820 | 0.238 | 0.697 | 0.423 | 0.589 |
| **Frontal_Inf_Oper_R2302** | **R** | 0.053 | -0.257 | 0.179 | -0.185 | -0.581 | -0.127 | -0.398 | -0.215 |
|  | **P** | 0.932 | 0.677 | 0.773 | 0.766 | 0.305 | 0.839 | 0.508 | 0.728 |
| **Frontal_Inf_Tri_L2311** | **R** | 0.031 | -0.291 | 0.164 | -0.216 | -0.554 | -0.168 | -0.392 | -0.277 |
|  | **P** | 0.960 | 0.635 | 0.792 | 0.728 | 0.332 | 0.787 | 0.514 | 0.652 |
| **Frontal_Inf_Tri_R2312** | **R** | 0.077 | -0.237 | 0.200 | -0.167 | -0.602 | -0.170 | -0.427 | -0.263 |
|  | **P** | 0.902 | 0.701 | 0.746 | 0.788 | 0.283 | 0.784 | 0.473 | 0.669 |
| **Frontal_Inf_Orb_L2321** | **R** | 0.043 | -0.270 | 0.155 | -0.213 | -0.611 | -0.240 | -0.422 | -0.327 |
|  | **P** | 0.945 | 0.660 | 0.804 | 0.730 | 0.273 | 0.698 | 0.479 | 0.591 |
| **Frontal_Inf_Orb_R2322** | **R** | 0.111 | -0.207 | 0.233 | -0.137 | -0.622 | -0.217 | -0.464 | -0.319 |
|  | **P** | 0.859 | 0.738 | 0.706 | 0.826 | 0.263 | 0.726 | 0.431 | 0.601 |
| **Rolandic_Oper_L2331** | **R** | 0.158 | -0.172 | 0.311 | -0.071 | -0.549 | -0.140 | -0.465 | -0.285 |
|  | **P** | 0.799 | 0.782 | 0.611 | 0.910 | 0.338 | 0.822 | 0.430 | 0.642 |
| **Rolandic_Oper_R2332** | **R** | 0.075 | -0.243 | 0.227 | -0.146 | -0.511 | -0.050 | -0.382 | -0.171 |
|  | **P** | 0.904 | 0.693 | 0.714 | 0.815 | 0.379 | 0.937 | 0.525 | 0.783 |
| **Supp_Motor_Area_L2401** | **R** | -0.127 | -0.432 | 0.024 | -0.346 | -0.312 | -0.085 | -0.209 | -0.230 |
|  | **P** | 0.838 | 0.467 | 0.970 | 0.569 | 0.609 | 0.892 | 0.736 | 0.710 |
| **Supp_Motor_Area_R2402** | **R** | 0.035 | -0.282 | 0.168 | -0.205 | -0.558 | -0.122 | -0.384 | -0.221 |
|  | **P** | 0.955 | 0.646 | 0.787 | 0.741 | 0.329 | 0.845 | 0.524 | 0.721 |
| **OIfactory_L2501** | **R** | 0.320 | -0.011 | 0.460 | 0.089 | -0.622 | -0.281 | -0.605 | -0.448 |
|  | **P** | 0.600 | 0.986 | 0.435 | 0.887 | 0.263 | 0.647 | 0.280 | 0.449 |
| **OIfactory_R2502** | **R** | 0.311 | -0.017 | 0.450 | 0.081 | -0.631 | -0.262 | -0.597 | -0.419 |
|  | **P** | 0.610 | 0.979 | 0.447 | 0.897 | 0.254 | 0.670 | 0.288 | 0.483 |
| **Frontal_Sup_Medial_L2601** | **R** | 0.139 | -0.191 | 0.293 | -0.089 | -0.468 | -0.217 | -0.445 | -0.397 |
|  | **P** | 0.824 | 0.758 | 0.632 | 0.887 | 0.427 | 0.726 | 0.453 | 0.508 |
| **Frontal_Sup_Medial_R2602** | **R** | 0.135 | -0.194 | 0.279 | -0.103 | -0.569 | -0.168 | -0.461 | -0.301 |
|  | **P** | 0.829 | 0.755 | 0.650 | 0.870 | 0.317 | 0.787 | 0.435 | 0.622 |
| **Frontal_Mid_Orb_L2611** | **R** | 0.147 | -0.076 | 0.289 | 0.034 | -0.072 | -0.172 | -0.271 | -0.406 |
|  | **P** | 0.813 | 0.903 | 0.637 | 0.957 | 0.909 | 0.781 | 0.660 | 0.498 |
| **Frontal_Mid_Orb_R2612** | **R** | 0.284 | -0.034 | 0.433 | 0.074 | -0.469 | -0.308 | -0.540 | -0.518 |
|  | **P** | 0.643 | 0.957 | 0.467 | 0.906 | 0.426 | 0.615 | 0.348 | 0.372 |
| **Rectus_L2701** | **R** | 0.117 | -0.035 | 0.221 | 0.049 | 0.047 | -0.186 | -0.180 | -0.392 |
|  | **P** | 0.852 | 0.956 | 0.721 | 0.937 | 0.940 | 0.765 | 0.773 | 0.514 |
| **Rectus_R2702** | **R** | 0.195 | -0.132 | 0.337 | -0.038 | -0.520 | -0.288 | -0.503 | -0.463 |
|  | **P** | 0.753 | 0.833 | 0.579 | 0.952 | 0.369 | 0.639 | 0.388 | 0.432 |
| **Insula_L3001** | **R** | 0.237 | -0.089 | 0.366 | -0.005 | -0.644 | -0.265 | -0.557 | -0.398 |
|  | **P** | 0.701 | 0.887 | 0.544 | 0.994 | 0.241 | 0.667 | 0.330 | 0.507 |
| **Insula_R3002** | **R** | 0.186 | -0.138 | 0.323 | -0.049 | -0.604 | -0.187 | -0.501 | -0.315 |
|  | **P** | 0.764 | 0.825 | 0.596 | 0.938 | 0.280 | 0.764 | 0.390 | 0.606 |
| **Cingulum_Ant_L4001** | **R** | 0.319 | -0.017 | 0.489 | 0.112 | -0.492 | -0.198 | -0.557 | -0.413 |
|  | **P** | 0.601 | 0.978 | 0.403 | 0.858 | 0.400 | 0.749 | 0.330 | 0.490 |
| **Cingulum_Ant_R4002** | **R** | 0.432 | 0.106 | 0.586 | 0.227 | -0.571 | -0.282 | -0.654 | -0.491 |
|  | **P** | 0.467 | 0.866 | 0.299 | 0.714 | 0.315 | 0.646 | 0.231 | 0.401 |
| **Cingulum_Mid_L4011** | **R** | -0.139 | -0.356 | 0.054 | -0.213 | 0.216 | 0.191 | 0.044 | -0.051 |
|  | **P** | 0.824 | 0.557 | 0.932 | 0.731 | 0.727 | 0.759 | 0.943 | 0.935 |
| **Cingulum_Mid_R4012** | **R** | 0.083 | -0.246 | 0.280 | -0.106 | -0.273 | -0.028 | -0.325 | -0.254 |
|  | **P** | 0.894 | 0.690 | 0.649 | 0.866 | 0.656 | 0.964 | 0.594 | 0.680 |
| **Cingulum_Post_L4021** | **R** | -0.273 | -0.539 | -0.088 | -0.421 | 0.027 | 0.158 | 0.036 | -0.028 |
|  | **P** | 0.657 | 0.349 | 0.888 | 0.481 | 0.966 | 0.800 | 0.954 | 0.964 |
| **Cingulum_Post_R4022** | **R** | -0.256 | -0.554 | -0.101 | -0.471 | -0.319 | 0.121 | -0.093 | 0.035 |
|  | **P** | 0.677 | 0.333 | 0.871 | 0.424 | 0.601 | 0.846 | 0.882 | 0.955 |
| **Hippocampus_L4101** | **R** | 0.156 | -0.169 | 0.293 | -0.082 | -0.596 | -0.182 | -0.480 | -0.307 |
|  | **P** | 0.803 | 0.786 | 0.633 | 0.895 | 0.289 | 0.769 | 0.413 | 0.615 |
| **Hippocampus_R4102** | **R** | 0.102 | -0.210 | 0.224 | -0.139 | -0.615 | -0.174 | -0.445 | -0.266 |
|  | **P** | 0.870 | 0.735 | 0.717 | 0.823 | 0.269 | 0.780 | 0.453 | 0.666 |
| **ParaHippocampal_L4111** | **R** | 0.108 | -0.200 | 0.214 | -0.144 | -0.659 | -0.239 | -0.469 | -0.317 |
|  | **P** | 0.863 | 0.747 | 0.730 | 0.818 | 0.227 | 0.698 | 0.425 | 0.603 |
| **ParaHippocampal_R4112** | **R** | 0.071 | -0.226 | 0.169 | -0.179 | -0.659 | -0.220 | -0.438 | -0.277 |
|  | **P** | 0.910 | 0.715 | 0.786 | 0.774 | 0.226 | 0.723 | 0.460 | 0.652 |
| **Amygdala_L4201** | **R** | 0.333 | 0.018 | 0.441 | 0.089 | -0.724 | -0.361 | -0.646 | -0.484 |
|  | **P** | 0.584 | 0.977 | 0.457 | 0.887 | 0.167 | 0.550 | 0.239 | 0.408 |
| **Amygdala_R4202** | **R** | 0.223 | -0.079 | 0.316 | -0.028 | -0.731 | -0.322 | -0.568 | -0.400 |
|  | **P** | 0.719 | 0.899 | 0.605 | 0.965 | 0.161 | 0.598 | 0.318 | 0.505 |
| **Calcarine_L5001** | **R** | 0.201 | -0.138 | 0.387 | -0.002 | -0.414 | -0.091 | -0.449 | -0.303 |
|  | **P** | 0.746 | 0.825 | 0.520 | 0.997 | 0.488 | 0.885 | 0.448 | 0.620 |
| **Calcarine_R5002** | **R** | 0.076 | -0.257 | 0.266 | -0.124 | -0.376 | 0.037 | -0.339 | -0.143 |
|  | **P** | 0.904 | 0.676 | 0.666 | 0.842 | 0.533 | 0.953 | 0.577 | 0.818 |
| **Cuneus_L5011** | **R** | -0.004 | -0.296 | 0.124 | -0.222 | -0.517 | -0.013 | -0.315 | -0.085 |
|  | **P** | 0.994 | 0.628 | 0.842 | 0.720 | 0.372 | 0.983 | 0.606 | 0.892 |
| **Cuneus_R5012** | **R** | 0.059 | -0.248 | 0.198 | -0.161 | -0.529 | -0.044 | -0.369 | -0.143 |
|  | **P** | 0.926 | 0.688 | 0.749 | 0.796 | 0.360 | 0.944 | 0.541 | 0.819 |
| **Lingual_L5021** | **R** | 0.233 | -0.059 | 0.330 | -0.001 | -0.704 | -0.251 | -0.547 | -0.326 |
|  | **P** | 0.706 | 0.925 | 0.587 | 0.999 | 0.185 | 0.684 | 0.340 | 0.593 |
| **Lingual_R5022** | **R** | 0.208 | -0.098 | 0.330 | -0.020 | -0.640 | -0.185 | -0.513 | -0.287 |
|  | **P** | 0.737 | 0.875 | 0.588 | 0.974 | 0.245 | 0.766 | 0.377 | 0.640 |
| **Occipital_Sup_L5101** | **R** | -0.026 | -0.315 | 0.088 | -0.256 | -0.558 | -0.070 | -0.322 | -0.123 |
|  | **P** | 0.967 | 0.606 | 0.888 | 0.677 | 0.328 | 0.911 | 0.597 | 0.844 |
| **Occipital_Sup_R5102** | **R** | 0.024 | -0.281 | 0.158 | -0.202 | -0.538 | -0.058 | -0.353 | -0.145 |
|  | **P** | 0.969 | 0.647 | 0.800 | 0.744 | 0.350 | 0.926 | 0.560 | 0.815 |
| **Occipital_Mid_L5201** | **R** | 0.021 | -0.294 | 0.153 | -0.218 | -0.552 | -0.112 | -0.371 | -0.207 |
|  | **P** | 0.974 | 0.631 | 0.806 | 0.724 | 0.334 | 0.858 | 0.539 | 0.738 |
| **Occipital_Mid_R5202** | **R** | 0.009 | -0.298 | 0.152 | -0.212 | -0.503 | -0.020 | -0.330 | -0.117 |
|  | **P** | 0.988 | 0.626 | 0.807 | 0.732 | 0.388 | 0.974 | 0.588 | 0.852 |
| **Occipital_Inf_L5301** | **R** | 0.049 | -0.273 | 0.188 | -0.191 | -0.549 | -0.133 | -0.395 | -0.245 |
|  | **P** | 0.937 | 0.656 | 0.762 | 0.758 | 0.338 | 0.831 | 0.510 | 0.691 |
| **Occipital_Inf_R5302** | **R** | -0.024 | -0.332 | 0.118 | -0.249 | -0.492 | -0.016 | -0.307 | -0.110 |
|  | **P** | 0.969 | 0.585 | 0.850 | 0.687 | 0.400 | 0.979 | 0.615 | 0.861 |
| **Fusiform_L5401** | **R** | 0.213 | -0.093 | 0.321 | -0.028 | -0.686 | -0.254 | -0.540 | -0.345 |
|  | **P** | 0.731 | 0.882 | 0.598 | 0.965 | 0.201 | 0.680 | 0.347 | 0.570 |
| **Fusiform_R5402** | **R** | 0.088 | -0.189 | 0.175 | -0.148 | -0.667 | -0.189 | -0.432 | -0.225 |
|  | **P** | 0.889 | 0.760 | 0.779 | 0.812 | 0.219 | 0.761 | 0.467 | 0.716 |
| **Postcentral_L6001** | **R** | -0.017 | -0.334 | 0.127 | -0.250 | -0.503 | -0.062 | -0.328 | -0.166 |
|  | **P** | 0.979 | 0.583 | 0.839 | 0.684 | 0.388 | 0.921 | 0.590 | 0.790 |
| **Postcentral_R6002** | **R** | -0.056 | -0.345 | 0.094 | -0.253 | -0.417 | 0.102 | -0.235 | 0.015 |
|  | **P** | 0.929 | 0.569 | 0.881 | 0.681 | 0.485 | 0.870 | 0.703 | 0.981 |
| **Parietal_Sup_L6101** | **R** | -0.131 | -0.440 | 0.017 | -0.358 | -0.426 | 0.022 | -0.220 | -0.071 |
|  | **P** | 0.834 | 0.458 | 0.978 | 0.554 | 0.475 | 0.973 | 0.722 | 0.910 |
| **Parietal_Sup_R6102** | **R** | -0.054 | -0.363 | 0.111 | -0.259 | -0.398 | 0.089 | -0.246 | -0.026 |
|  | **P** | 0.932 | 0.548 | 0.859 | 0.674 | 0.507 | 0.887 | 0.691 | 0.967 |
| **Parietal_Inf_L6201** | **R** | -0.005 | -0.312 | 0.117 | -0.248 | -0.569 | -0.127 | -0.359 | -0.205 |
|  | **P** | 0.994 | 0.609 | 0.852 | 0.687 | 0.317 | 0.838 | 0.553 | 0.741 |
| **Parietal_Inf_R6202** | **R** | -0.042 | -0.345 | 0.102 | -0.259 | -0.468 | 0.023 | -0.279 | -0.066 |
|  | **P** | 0.946 | 0.569 | 0.870 | 0.673 | 0.427 | 0.970 | 0.649 | 0.916 |
| **SupraMarginal_L6211** | **R** | 0.044 | -0.277 | 0.178 | -0.200 | -0.560 | -0.153 | -0.398 | -0.261 |
|  | **P** | 0.944 | 0.652 | 0.775 | 0.747 | 0.326 | 0.805 | 0.507 | 0.672 |
| **SupraMarginal_R6212** | **R** | -0.002 | -0.309 | 0.134 | -0.230 | -0.520 | -0.044 | -0.331 | -0.133 |
|  | **P** | 0.997 | 0.613 | 0.830 | 0.710 | 0.369 | 0.944 | 0.586 | 0.832 |
| **Angular_L6221** | **R** | 0.021 | -0.286 | 0.133 | -0.229 | -0.607 | -0.184 | -0.394 | -0.257 |
|  | **P** | 0.973 | 0.641 | 0.832 | 0.710 | 0.278 | 0.767 | 0.512 | 0.677 |
| **Angular_R6222** | **R** | -0.001 | -0.315 | 0.145 | -0.227 | -0.498 | -0.034 | -0.329 | -0.138 |
|  | **P** | 0.999 | 0.606 | 0.816 | 0.713 | 0.393 | 0.956 | 0.589 | 0.825 |
| **Precuneus_L6301** | **R** | -0.120 | -0.443 | 0.051 | -0.340 | -0.337 | 0.017 | -0.212 | -0.127 |
|  | **P** | 0.847 | 0.454 | 0.935 | 0.576 | 0.579 | 0.978 | 0.732 | 0.839 |
| **Precuneus_R6302** | **R** | 0.005 | -0.329 | 0.181 | -0.214 | -0.375 | -0.051 | -0.313 | -0.224 |
|  | **P** | 0.994 | 0.589 | 0.771 | 0.729 | 0.534 | 0.936 | 0.608 | 0.717 |
| **Paracentral_Lobule_L6401** | **R** | -0.077 | -0.370 | 0.045 | -0.308 | -0.519 | -0.040 | -0.279 | -0.099 |
|  | **P** | 0.903 | 0.539 | 0.942 | 0.614 | 0.370 | 0.949 | 0.649 | 0.874 |
| **Paracentral_Lobule_R6402** | **R** | 0.023 | -0.274 | 0.146 | -0.205 | -0.560 | -0.074 | -0.357 | -0.146 |
|  | **P** | 0.970 | 0.655 | 0.815 | 0.741 | 0.326 | 0.906 | 0.555 | 0.815 |
| **Caudate_L7001** | **R** | 0.223 | -0.059 | 0.396 | 0.075 | -0.375 | 0.107 | -0.382 | -0.053 |
|  | **P** | 0.718 | 0.925 | 0.509 | 0.904 | 0.534 | 0.864 | 0.526 | 0.933 |
| **Caudate_R7002** | **R** | 0.404 | 0.132 | 0.564 | 0.268 | -0.436 | -0.005 | -0.518 | -0.183 |
|  | **P** | 0.500 | 0.832 | 0.322 | 0.663 | 0.463 | 0.994 | 0.372 | 0.769 |
| **Putamen_L7011** | **R** | 0.641 | 0.547 | 0.771 | 0.701 | 0.110 | -0.025 | -0.404 | -0.306 |
|  | **P** | 0.244 | 0.340 | 0.127 | 0.187 | 0.860 | 0.968 | 0.499 | 0.616 |
| **Putamen_R7012** | **R** | 0.594 | 0.309 | 0.756 | 0.455 | -0.469 | -0.190 | -0.679 | -0.429 |
|  | **P** | 0.291 | 0.613 | 0.140 | 0.441 | 0.426 | 0.760 | 0.207 | 0.472 |
| **Pallidum_L7021** | **R** | 0.409 | 0.111 | 0.537 | 0.211 | -0.638 | -0.215 | -0.629 | -0.359 |
|  | **P** | 0.495 | 0.859 | 0.351 | 0.734 | 0.247 | 0.728 | 0.255 | 0.553 |
| **Pallidum_R7022** | **R** | 0.350 | 0.065 | 0.454 | 0.139 | -0.692 | -0.241 | -0.606 | -0.339 |
|  | **P** | 0.564 | 0.917 | 0.443 | 0.823 | 0.196 | 0.696 | 0.279 | 0.576 |
| **Thalamus_L7101** | **R** | -0.004 | -0.246 | 0.130 | -0.157 | -0.371 | 0.179 | -0.210 | 0.115 |
|  | **P** | 0.995 | 0.691 | 0.835 | 0.801 | 0.539 | 0.773 | 0.734 | 0.854 |
| **Thalamus_R7102** | **R** | 0.184 | -0.080 | 0.313 | 0.013 | -0.500 | 0.018 | -0.398 | -0.074 |
|  | **P** | 0.767 | 0.899 | 0.608 | 0.984 | 0.391 | 0.978 | 0.507 | 0.905 |
| **Heschl_L8101** | **R** | 0.205 | -0.129 | 0.363 | -0.020 | -0.543 | -0.161 | -0.497 | -0.325 |
|  | **P** | 0.740 | 0.836 | 0.548 | 0.974 | 0.345 | 0.796 | 0.394 | 0.593 |
| **Heschl_R8102** | **R** | 0.161 | -0.168 | 0.330 | -0.049 | -0.484 | -0.052 | -0.431 | -0.213 |
|  | **P** | 0.796 | 0.788 | 0.587 | 0.937 | 0.409 | 0.934 | 0.468 | 0.731 |
| **Temporal_Sup_L8111** | **R** | 0.217 | -0.120 | 0.373 | -0.012 | -0.541 | -0.200 | -0.512 | -0.374 |
|  | **P** | 0.726 | 0.847 | 0.536 | 0.984 | 0.346 | 0.747 | 0.378 | 0.535 |
| **Temporal_Sup_R8112** | **R** | 0.110 | -0.211 | 0.258 | -0.115 | -0.537 | -0.086 | -0.418 | -0.211 |
|  | **P** | 0.861 | 0.733 | 0.675 | 0.854 | 0.351 | 0.890 | 0.484 | 0.734 |
| **Temporal_Pole_Sup_L8121** | **R** | 0.179 | -0.122 | 0.258 | -0.087 | -0.722 | -0.429 | -0.559 | -0.510 |
|  | **P** | 0.774 | 0.844 | 0.675 | 0.889 | 0.168 | 0.471 | 0.327 | 0.380 |
| **Temporal_Pole_Sup_R8122** | **R** | 0.092 | -0.225 | 0.220 | -0.150 | -0.597 | -0.167 | -0.436 | -0.268 |
|  | **P** | 0.883 | 0.716 | 0.722 | 0.810 | 0.288 | 0.788 | 0.463 | 0.663 |
| **Temporal_Mid_L8201** | **R** | 0.044 | -0.288 | 0.209 | -0.181 | -0.413 | -0.121 | -0.358 | -0.294 |
|  | **P** | 0.944 | 0.638 | 0.735 | 0.771 | 0.490 | 0.846 | 0.554 | 0.631 |
| **Temporal_Mid_R8202** | **R** | 0.072 | -0.256 | 0.233 | -0.151 | -0.486 | -0.052 | -0.380 | -0.192 |
|  | **P** | 0.908 | 0.678 | 0.706 | 0.809 | 0.406 | 0.934 | 0.528 | 0.757 |
| **Temporal_Pole_Mid_L8211** | **R** | 0.015 | -0.268 | 0.085 | -0.249 | -0.689 | -0.312 | -0.422 | -0.340 |
|  | **P** | 0.981 | 0.663 | 0.892 | 0.686 | 0.198 | 0.610 | 0.479 | 0.575 |
| **Temporal_Pole_Mid_R8212** | **R** | -0.007 | -0.282 | 0.072 | -0.254 | -0.652 | -0.196 | -0.373 | -0.216 |
|  | **P** | 0.991 | 0.645 | 0.908 | 0.680 | 0.233 | 0.752 | 0.536 | 0.727 |
| **Temporal_Inf_L8301** | **R** | 0.096 | -0.220 | 0.213 | -0.156 | -0.629 | -0.234 | -0.459 | -0.329 |
|  | **P** | 0.878 | 0.722 | 0.731 | 0.802 | 0.256 | 0.705 | 0.437 | 0.589 |
| **Temporal_Inf_R8302** | **R** | 0.055 | -0.248 | 0.171 | -0.184 | -0.602 | -0.139 | -0.402 | -0.213 |
|  | **P** | 0.931 | 0.688 | 0.783 | 0.767 | 0.283 | 0.823 | 0.503 | 0.731 |
| **Precentral_L2001** | **R** | 0.333 | 0.018 | 0.441 | 0.089 | -0.724 | -0.361 | -0.646 | -0.484 |
|  | **P** | 0.584 | 0.977 | 0.457 | 0.887 | 0.167 | 0.550 | 0.239 | 0.408 |
| **Precentral_R2002** | **R** | 0.223 | -0.079 | 0.316 | -0.028 | -0.731 | -0.322 | -0.568 | -0.400 |
|  | **P** | 0.719 | 0.899 | 0.605 | 0.965 | 0.161 | 0.598 | 0.318 | 0.505 |
| **Frontal_Sup_L2101** | **R** | 0.223 | -0.059 | 0.396 | 0.075 | -0.375 | 0.107 | -0.382 | -0.053 |
|  | **P** | 0.718 | 0.925 | 0.509 | 0.904 | 0.534 | 0.864 | 0.526 | 0.933 |
| **Frontal_Sup_R2102** | **R** | 0.404 | 0.132 | 0.564 | 0.268 | -0.436 | -0.005 | -0.518 | -0.183 |
|  | **P** | 0.500 | 0.832 | 0.322 | 0.663 | 0.463 | 0.994 | 0.372 | 0.769 |
| **Frontal_Sup_Orb_L2111** | **R** | 0.641 | 0.547 | 0.771 | 0.701 | 0.110 | -0.025 | -0.404 | -0.306 |
|  | **P** | 0.244 | 0.340 | 0.127 | 0.187 | 0.860 | 0.968 | 0.499 | 0.616 |
| **Frontal_Sup_Orb_R2112** | **R** | 0.594 | 0.309 | 0.756 | 0.455 | -0.469 | -0.190 | -0.679 | -0.429 |
|  | **P** | 0.291 | 0.613 | 0.140 | 0.441 | 0.426 | 0.760 | 0.207 | 0.472 |
